# NRF2 Loss Accentuates Parkinsonian Pathology and Behavioral Dysfunction in Human α-Synuclein Overexpressing Mice

**DOI:** 10.14336/AD.2021.0511

**Published:** 2021-07-01

**Authors:** Annadurai Anandhan, Nhat Nguyen, Arjun Syal, Luke A Dreher, Matthew Dodson, Donna D Zhang, Lalitha Madhavan

**Affiliations:** ^1^Pharmacology and Toxicology, University of Arizona, Tucson, AZ, USA.; ^2^Department of Neurology, University of Arizona, Tucson, AZ, USA.; ^3^Physiology Undergraduate Program, Tucson, AZ, USA.; ^4^Neuroscience and Cognitive Science Undergraduate Program, Tucson, AZ, USA.; ^5^Ecology and Evolutionary Biology Undergraduate Program, Tucson, AZ, USA.; ^6^Evelyn F McKnight Brain Institute and Bio5 Institute, University of Arizona, Tucson, AZ, USA.

**Keywords:** NRF2, α-Synuclein, Parkinson’s disease, Motor dysfunction, Oxidative stress, Proteotoxic stress

## Abstract

Nuclear factor (erythroid-derived 2)-like 2 (NRF2) is a central regulator of cellular stress responses and its transcriptional activation promotes multiple cellular defense and survival mechanisms. The loss of NRF2 has been shown to increase oxidative and proteotoxic stress, two key pathological features of neurodegenerative disorders such as Parkinson’s disease (PD). Moreover, compromised redox homeostasis and protein quality control can cause the accumulation of pathogenic proteins, including alpha-synuclein (α-Syn) which plays a key role in PD. However, despite this link, the precise mechanisms by which NRF2 may regulate PD pathology is not clear. In this study, we generated a humanized mouse model to study the importance of NRF2 in the context of α-Syn-driven neuropathology in PD. Specifically, we developed NRF2 knockout and wild-type mice that overexpress human α-Syn (hα-Syn^+^/Nrf2^-/-^ and hα-Syn^+^/Nrf2^+/+^ respectively) and tested changes in their behavior through nest building, challenging beam, and open field tests at three months of age. Cellular and molecular alterations in α-Syn, including phosphorylation and subsequent oligomerization, as well as changes in oxidative stress, inflammation, and autophagy were also assessed across multiple brain regions. It was observed that although monomeric α-Syn levels did not change, compared to their wild-type counterparts, hα-Syn^+^/Nrf2^-/-^ mice exhibited increased phosphorylation and oligomerization of α-Syn. This was associated with a loss of tyrosine hydroxylase expressing dopaminergic neurons in the substantia nigra, and more pronounced behavioral deficits reminiscent of early-stage PD, in the hα-Syn^+^/Nrf2^-/-^ mice. Furthermore, hα-Syn^+^/Nrf2^-/-^ mice showed significantly amplified oxidative stress, greater expression of inflammatory markers, and signs of increased autophagic burden, especially in the midbrain, striatum and cortical brain regions. These results support an important role for NRF2, early in PD progression. More broadly, it indicates NRF2 biology as fundamental to PD pathogenesis and suggests that targeting NRF2 activation may delay the onset and progression of PD.

Nuclear factor (erythroid-derived 2)-like 2 (or NRF2) is a transcription factor that is critical to the cell’s homeostatic mechanism and functions to promote a broad range of cell survival processes in response to stress [1-3]. Under basal conditions, NRF2 is bound by its negative regulator Kelch-like ECH associated protein 1 (KEAP1) in the cytoplasm, that constantly targets it for ubiquitination and proteasomal degradation [4, 5]. However, under stress, specific modifications to the KEAP1-NRF2 complex occur, that allow NRF2 to stabilize and subsequently migrate to the nucleus to stimulate multiple cellular defense genes. In fact, NRF2 can regulate the expression of a vast number of genes bearing an antioxidant response element (ARE), and activate targets involved in nearly every facet of cell signaling and metabolism, including redox regulation, proteostasis, DNA repair, prevention of apoptosis, drug/xenobiotic metabolism, iron homeostasis, lipid and carbohydrate catabolism, and mitochondrial function [3]. Based on its control of a host of transcriptional responses, loss of proper NRF2 function can result in maladaptive effects across several pathological contexts [6]. For instance, the decreased expression of NRF2 is associated with many of the key pathogenic hallmarks of aging and age-related pathologies, including increased oxidative stress and inflammation, disruption of proteostasis, genomic instability, and mitochondrial dysfunction, among others [7].

Alterations in NRF2 signaling have been linked to neurodegeneration in Parkinson’s disease (PD) [8-10]. PD is the second most common age-related neurodegenerative disorder and is characterized by chronic progressive motor deficits and neurocognitive decline [11]. Neuropathologically, the pathognomonic features of PD include the loss of dopaminergic (DA) neurons in the substantia nigra pars compacta (SNpc), and Lewy pathology, which is the abnormal aggregation of the protein alpha-synuclein (α-Syn) in degenerating neurons [12-14]. In its native form α-Syn has diverse neural functions, however when misfolded, it forms intracellular inclusions within the cell body (Lewy bodies) and processes (Lewy neurites) which cause cellular toxicity [15, 16]. This abnormal α-Syn accumulation is associated with changes such as oxidative stress, inflammation and altered proteostasis, which are other key aspects of PD [17-21]. In this regard, it has been shown that NRF2 mitigates neurodegenerative phenotypes in cellular and animal models of PD, including acute virally induced α-Syn models [22-24]. Conversely, acute α-Syn-mediated PD pathology is heightened in the absence of NRF2 [25]. Interestingly, it has also been noted that NRF2 is primarily cytosolic in nigral DA neurons of healthy individuals as compared to age-matched PD patients where it is found in the nucleus [10]. Moreover, a number of NRF2 target genes, including NADPH quinone oxidoreductase 1 (*NQO1*) and heme oxygenase-1 (*HMOX1*), have been shown to be elevated in PD brains [8, 26-28]. These studies support the notion that NRF2 status can dictate the pathogenic effects of α-Syn and influence PD pathogenesis. Nevertheless, the precise role of NRF2 loss in endogenous development of α-Syn pathology in PD, and its associated cellular, molecular, and behavioral effects remains elusive.

Here, we use a genetic approach to address this question. Specifically, we generated novel mice by cross breeding previously established Nrf2 knockout (Nrf2^-/-^) or wild-type (Nrf2^+/+^) mice [29], with mice overexpressing full length human wild-type α-Syn under the Thy-1 promoter (Thy1-α-Syn mice) [30]. The Thy1-α-Syn mice recapitulate many progressive features of sporadic PD seen in patients with the disease [30]. Using the newly established hα-Syn/Nrf2 mouse model, we comprehensively examined the involvement of NRF2 in the development of PD pathology and behavioral deficits. We found that NRF2 loss significantly worsens sensorimotor function, increases α-Syn aggregation, oxidative stress, inflammation, and autophagic burden, and induces DA neuron degeneration, as early as 3 mos of age. To our knowledge, these data are the first to explicate NRF2’s involvement in promoting α-Syn pathology and its functional effects in a translationally relevant representation of PD onset and progression.

## MATERIALS AND METHODS

### Animals

**Figure 1.**
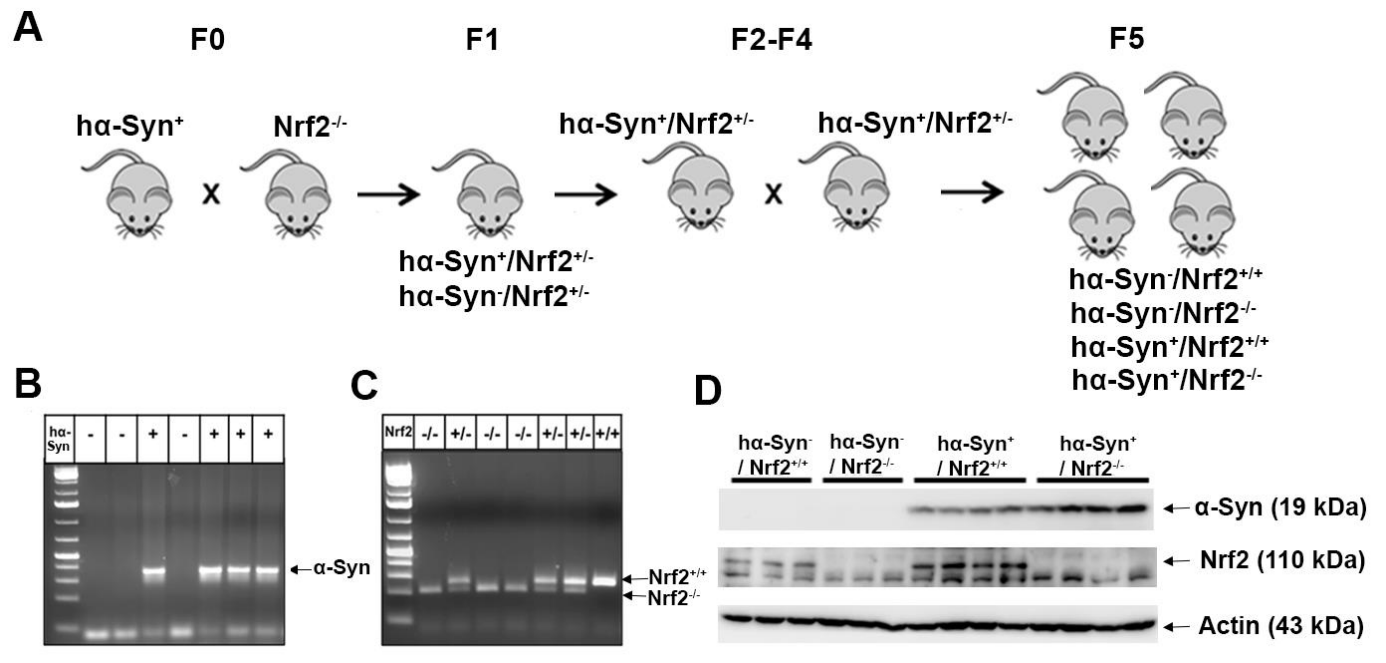
Generation of a novel humanized α-Syn/NRF2 mouse model of PD. (A) Mice overexpressing human wild-type α-Syn (hα-Syn^+^) were initially cross bred with Nrf2 knockout (Nrf2^-/-^) mice to result in *hα-Syn^+^/Nrf2^+/-^* and *hα-Syn^-^/Nrf2^+/-^* mouse strains. The *hα-Syn^+^/Nrf2^+/-^* mice were further crossed to finally generate four genotypes: *hα-Syn^+^/Nrf2^+/+^*, *hα-Syn^+^/Nrf2^-/-^*, *hα-Syn^-^/Nrf2^+/+^* and *hα-Syn^-^/Nrf2^-/-^* strains. Mouse genotypes were confirmed by PCR (B and C) of tail DNA and western blotting (D).

Hemizygous human Thy1-α-Syn mice (hα-Syn^+^; C57Bl6/DBA background; founder breeder pairs were originally provided by Dr Marie-Francoise Chesselet, UCLA) [30], Nrf2 knockout (Nrf2^-/-^) [29], and wild-type (Nrf2^+/+^) mice were maintained in the University of Arizona Animal Care Facility. Mice were handled according to the rules and regulations of the NIH and Institutional Guidelines on the Care and Use of Laboratory Animals. All experimental protocols were approved by the University of Arizona Institutional Animal Care and Use Committee. The mice were housed under a reverse 12-hour light-dark cycle condition with food and water available *ad libitum*. The hemizygous human Thy1-α-Syn mice were crossed with Nrf2^-/-^ mice to generate hα-Syn^-^/Nrf2^+/-^ and hα-Syn^+^/Nrf2^+/-^ mice. Heterozygous hα-Syn^+^/Nrf2^+/-^ were subsequently cross bred to generate four genotypes: hα-Syn^-^/Nrf2^+/+^, hα-Syn^-^/Nrf2^-/-^, hα-Syn^+^/Nrf2^+/+^ and hα-Syn^+^/Nrf2^-/-^. The breeding strategy is depicted in Figure 1. Male littermates were used in the study, and the genotypes of all mice were verified with PCR analysis of tail DNA.

### Experimental Design

All four genotypes of mice produced were maintained until 3 mos of age at which point they were subjected to behavioral tests. Subsequently, animals were sacrificed at ~3.5 mos of age and brains processed for histological and molecular studies. The number of animals per group were as follows: hα-Syn^-^/Nrf2^+/+^ (*n* = 16), hα-Syn^-^/Nrf2^-/-^ (*n* = 14), hα-Syn^+^/Nrf2^+/+^ (*n* = 17) and hα-Syn^+^/Nrf2^-/-^ (*n* = 16).

For histological studies, mice were sacrificed using sodium pentobarbital (60 mg/kg) and perfusion with 4% paraformaldehyde (PFA). Subsequently, brains were extracted and post-fixed in 4% PFA, sunk through a 30% sucrose solution, and sectioned (30 μm) in the coronal plane on a freezing sliding microtome. For molecular studies, animals were sacrificed using sodium pentobarbital (60 mg/kg), brains extracted and subjected to microdissection on ice to isolate tissues from the midbrain, striatum, cortex and hippocampus. The dissected tissues were then snap frozen in liquid nitrogen for later molecular analyses.

### Behavioral analyses

#### Nest Building Task

Nest building is a natural motor behavior requiring the use of orofacial and forelimb movements that can be used to assess PD relevant sensorimotor function in rodents [31]. This task tests the ability of the animal to retrieve and use woven cotton pads (nestlets) placed in their cage’s feeding bin. Once the nestlets are grasped and placed into the cage, the animals pull the nesting material apart with their forelimbs and teeth, breakdown the cotton in their mouths, and incorporate it into their bedding. Thus forelimb dexterity and fine motor behavior can be measured with this task. Briefly, mice are transferred to individual testing cages approximately 1 hr before the dark phase and weighed nestlets are placed in the feeder bin of the cage (5 nestlets, ~12g/cage). The amount of nestlets that were retrieved (nestlet pulldown) and broken down (nestlet usage) for nest building was measured every 12 hours over a 72 hour period (12, 24, 36, 48, 60 and 72 hours) by weighing the unused material in the feeder and inside the cage [31]. Data were presented as percent of nestlet pulldown and percent used as normalized to control (hα-Syn^-^/Nrf2^+/+^).

#### Challenging Beam Task

Mice were subjected to a challenging beam task to test motor coordination and agility [31, 32]. A wooden beam consisting of four continuous sections (25 cm in length each, 1m total length), which incrementally narrows from a starting width of 3.5 cm to an ending width of 0.5 cm, was used. The widest segment of the beam served as a loading platform for the animals and the narrowest end connected into the home cage. Mice received 2 days of training before testing. During the training phase, animals were brought to the behavior room at the start of their dark cycle and acclimated to the testing room for ~2 hrs each day. The animal was placed on the beam at its widest point and trained to move across the beam towards the narrowest part and into the home cage. Testing started on day 3 when mice underwent 5 unassisted trials on the beam. To increase difficulty, a mesh grid (1 cm^2^ grid) of corresponding width was placed over the beam surface leaving an ~1 cm space between the grid and the beam surface to allow adequate visualization of the mice’s paws as they walked. Each trial was videotaped, then viewed and rated in slow motion for foot slip errors, number of steps made by each animal to cross the beam, and time taken to traverse the beam, by an investigator blind to the mouse genotype. A foot slip error was counted when, during a forward movement, a limb (forelimb or hindlimb) slid through the grid and was visible between the grid and the beam surface. By scoring each limb slip individually, the severity of the error could be measured. The trial ended when an animal fell off the beam, reached the maximum allowed time (60 sec), or traversed the full distance [33]. Errors per step, time to traverse, and number of steps were calculated as the average across all five trials.

#### Open field task

The open field test was used to determine spontaneous exploratory activity of the mice [34]. Activity was assessed in an open arena (60 wide x 60 long x 60 cm high) with a central box drawn in the middle of the field floor (30 x 30 cm center). Animals were tested within the first 2-4 h of the dark cycle after being habituated to the testing room for 15 min. Mice were placed individually in the center of the open field (30 cm square) and their movement monitored (videotaped) for 15 min. Videos were analyzed for the following parameters: time spent moving, distance traveled, number of times the central box is encroached (center entries), time spent in central box (center time), number of rears, and time spent grooming [35].

### Western Blotting

Brain tissue from the four specified regions was homogenized using the TissueLyser II (QIAGEN, Germantown, MD) in 10 vol. (w/v) of cold RIPA buffer (25?mM Tris-HCl, pH 7.6, 150?mM NaCl, 1% Triton X-100, 1% sodium deoxycholate, 0.1% SDS) supplemented with 1 mM phenylmethylsulfonyl fluoride (PMSF) and a phosphatase inhibitor cocktail (PIC). Tissues were then sonicated for 10 s to generate *total cell lysates*. For isolating soluble and insoluble protein fractions, the *total cell lysates* were divided into Triton X-100 soluble and insoluble fractions by adding Triton X-100 (final concentration 1%) and incubating for 30 min on ice followed by centrifugation (15,000×*g*, 60 min, 4°C). The supernatant was designated as the *Triton X-100 soluble* fraction. The pellet was dissolved in lysis buffer containing 2% SDS and sonicated for 10 sec. This fraction was designated to be *Triton X-100 insoluble*. The protein concentration in the different lysates was determined via the bicinchoninic acid method (BCA, Thermo/Pierce, Waltham, MA). Lysates were then boiled, sonicated, and resolved by SDS-PAGE, and membranes were subjected to appropriate antibodies at 4°C for overnight. Then membranes were incubated with anti-mouse or anti-rabbit horseradish peroxidase (HRP) conjugated secondary antibodies (1:3000, Sigma Aldrich, St. Louis, MO) for 1 hr. All immunoblot images were taken using the Azure Biosystems c600 (Azure cSeries Advanced Imaging Systems, Dublin, CA). Relative densitometry analysis of western blots was performed using the ImageJ Program (National Institutes of Health, http://rsb.info.nih.gov/ij). For the densitometric quantification of α-Syn oligomers, all oligomeric bands were quantified and normalized to monomeric α-Syn.

The primary antibodies applied were: anti-NRF2 (1:1000, sc-13032), anti-COX-2 (1:1000, sc-166475), anti-iNOS2 (1:1000, sc-7271) from Santa Cruz Biotechnology, Dallas, TX. Anti-phospho Syn S129 (1:500, ab-59264), and anti-4-HNE (1:2000, ab-46545) from Abcam, Cambridge, UK. Anti-α-Syn (1:1000, AHB0261) from Life Technologies, Carlsbad, CA. α-Syn (clone 42, 1:1000, 610787) from BD Biosciences, San Jose, CA. Anti-LC3 (1:2000, L7543) and anti-β-actin (1:5000, A2066) from Sigma, Aldrich, St. Louis, MO. Anti-SQSTM1 (p62, 1:2000, H00008878-M01) from Abnova, Taipei, Taiwan. Anti-β-tubulin (1:2000, 2146S) from Cell Signaling, Danvers, MA.

### Immunohistochemistry

Immunohistochemistry was performed using previously established methods [36, 37]. Briefly, sections were blocked [10% normal goat serum, 0.5% Triton-X-100 in Tris buffered saline (TBS, pH 7.4)] and incubated in primary antibodies, anti-α-Syn (clone 42, 1:500 - 610787, BD Biosciences, San Jose, CA), anti-phospho α-Syn S129 (1:300 - ab-59264, Abcam, Cambridge, UK), anti-tyrosine hydroxylase (1:4000 - MAB318, Chemicon Temecula, CA) and anti-Iba1 (1:500 - 019-19741 Wako Chemicals, Richmond, VA) overnight at room temperature (RT). Primary antibodies were detected in a 2 hr incubation at RT with secondary antibodies coupled to fluorochromes Alexa 488 or 555 (Life Technologies-Molecular Probes, Grand Island, NY) and counterstained with 4',6'-diamidino-2-phenylindole, dihydrochloride (DAPI, Life Technologies). Alternatively, primaries were treated with biotinylated secondary antibodies (Vector Laboratories, Burlingame, CA) followed by ABC reagent (Vector Laboratories) and exposure to 3'-Diaminobenzidine (DAB: Sigma Aldrich, Saint Louis, MO). Control conditions constituted the deletion of the primary antibody or secondary antibody and the inclusion of relevant isotype specific antibodies and sera instead of the omitted antibodies. Sections probed with α-Syn and phospho α-Syn were counterstained with Hematoxylin.

### Stereology and Quantification

#### Stereology

Stereological probes were applied using a Zeiss Imager M2 microscope (Carl Zeiss, Jena, Germany) equipped with StereoInvestigator software (v2019.1.3; MBF Bioscience, VT, USA) according to previously published methods [37, 38]. Using the optical fractionator probe, Tyrosine Hydroxylase (TH) positive cells were counted in sections 480 μm apart using a grid size of 170 X 100 μm and counting frame size of 75 X 75 μm. Contours were drawn around the region of interest at 10X magnification, and cells were counted under a 63X oil immersion objective. Guard zones were set at 2 μm each at the top and bottom of the section, and the counting frame was lowered at 1-2 μm interludes and each cell in focus was marked. The Gundersen method for calculating the coefficient of error was used to estimate the accuracy of the optical fractionator results. Coefficients obtained were generally less than 0.1.

#### Other cellular quantifications

For the quantification of TH immunoreactivity in the striatum, 10x images were captured and the optical density (OD) of both hemispheres was measured using Image J software (NIH Image, Bethesda, MD) in 30 μm thick coronal sections (1:12 series, 4 sections per animal, n=6 animals/group). Quantification of Iba1 immunoreactivity was conducted on 40x confocal images (30 μm thick coronal sections, 1:6 series, 4 sections per animal, n=3 animals/group). The mean Iba1 fluorescence intensity was measured by Image J. The number of TH^+^/phospho-Syn^+^ cells were counted in 40x images (30 μm thick coronal sections, 1:6 series, 4 sections per animal, n=3 animals/group). Data was expressed as mean ± SEM of the total number of cells obtained across the sections counted in each experimental group.

### Real-Time qRT-PCR

Total mRNA was extracted using TRIzol (Invitrogen, Carlsbad, CA) according to the manufacturer’s instructions. cDNA was then synthesized using 2 μg of mRNA and a Transcriptase first-strand cDNA synthesis kit (Promega, Madison, WI). Real-Time qPCR to detect p62 was performed on a LightCycler® 480 System (Roche Life Science, Penzberg, Germany) using PowerUp SYBR Green Master Mix (Thermo Fisher Scientific, Waltham, MA). Actin was used as an internal control. The reaction conditions were as follows; UDG activation 50°C (2 min), Dual-Lock™ DNA polymerase 95 °C (2 min), Denaturation 95 °C (15 sec), Annealing 55-60°C (15 sec), Extension 72°C (1 min) and 40 cycles. All experiments were performed in triplicate. Relative expression levels were calculated using the 2?-?ΔΔCT method and primer sequences (5′-3′) were as follows:

mouse-p62-Forward 5’-GCTGCCCTATACCCACATC T-3’ mouse-p62- Reverse 5’-CGCCTTCATCCGAGA AAC-3’ mouse-Actin-Forward 5'-AAGGCCAACCGT GAAAAGAT-3' mouse-Actin-Reverse 5'-GTGGTACG ACCAGAGGCATAC-3'.

### Electron Paramagnetic Resonance (EPR)

EPR was performed as described previously [39]. Briefly, brain tissues were dissected (midbrain, striatum, hippocampus and cortex), and incubated with spin trap in the presence of metal chelators (200 µM cyclic hydroxylamine 1-hydroxy-. 3-methoxycarbonyl-2,2,5,5-tetramethylpyrrolidine [CMH], 25 µM deferoxamine [DF], and 5 µM diethyldithiocarbamate [DETC] in filtered 20 mM KREBS-HEPES buffer) (Noxygen) for 30 min prior to measurement. Buffer was then collected, and changes in CMH oxidation were measured for 15 min using the e-scanM Multipurpose Bench-top EPR system (Noxygen Science and Transfer Diagnostics GmbH). The relative production of reactive oxygen species was represented as the nanomolar concentration of oxidized spin trap divided by the time of trap incubation normalized to the total milligrams of tissue, then data were normalized to control.

### Transmission Electron Microscopy

Brains were extracted and four regions, namely the midbrain, striatum, hippocampus and cortex, were microdissected. Tissue slices for transmission (TEM) electron microscopy were fixed in 2.5% glutaraldehyde + 2% PFA in 0.1 M piperazine-N,N′-bis (2-ethanesulfonic acid) or PIPES buffer (pH 7.4) for 1 hr at RT or at 4°C overnight. Samples were post-fixed in 1% osmium tetroxide in PIPES buffer for 1 hr following a wash in 0.05 M PIPES buffer + 0.05 M Glycine and 2 X 10 min washes in 0.1 M PIPES. Following two more 10 min washes in deionized water (DIW), samples for TEM were block stained in 2% aqueous uranyl acetate, washed in DIW, dehydrated through a graded series of alcohols, infiltrated with 1:1 alcohol and Spurr’s resin overnight, and then embedded in 100% Spurr’s resin overnight at 60°C. Sections were viewed using a FEI Tecnai Spirit electron microscope (FEI Company, Hillsboro, OR) operated at 100 kV. 8-bit TIFF images were captured through an AMT 4 Mpixel camera. Morphometric measurements were conducted in digital images using Image J (NIH) software. Number of autophagic vacuoles per cell profile (16-20 cell profiles) across a minimum of 10 micrographs per animal were counted using standard criteria [20, 40]. Vesicles were classified as autophagic vacuoles (autophagosomes or autolysosomes) if they met two or more of the following criteria: for autophagosomes they should have a completely or partially visible double membrane, absence of ribosomes in the outer membrane, identifiable organelles or parts of organelles in their lumen, and luminal density comparable to the surrounding cytosol; for autolysosomes they should have similar size but with less than 40% of membrane visible as double, cargo in the lumen is not identifiable as particular organelles and/or amorphous material, and luminal density below that of the surrounding cytosol.

### Light and Confocal Microscopy

A Zeiss M2 Imager microscope (Carl Zeiss, Jena, Germany) connected to an AxioCam Mrc digital camera was used for brightfield microscopy (α-Syn, phospho-α-Syn and TH). Images were collected through a 63X lens for the α-Syn and phospho-α-Syn images, and a 20X lens for the TH images, using Microlucida software (v2019.1.3; MBF Bioscience, VT, USA). Fluorescence analysis was performed for α-Syn and Iba1 using a Leica SP5-II confocal microscope (Leica Microsystems, Wetzlar, Germany). 40X and 60X lenses were used, and Z sectioning was performed at 1-2 μm intervals in order to verify the co-localization of markers. Image extraction and analysis was conducted via the Leica LAS software (LAS AF 2.7.3.9723).

### Statistical analysis

GraphPad Prism 8 software (San Diego, CA) was used for statistical analyses. For comparing two groups, unpaired *t* tests were used. For comparisons between three or more groups, one-way analysis of variance (ANOVA) followed by Tukey’s post-hoc test for multiple comparisons between treatment groups was conducted. For analyzing the nest building behavior, a two-way repeated measures ANOVA was performed using the Sigma Plot 14 (San Jose, CA) software. Differences were accepted as significant at p < 0.05. Statistical details of each experiment are provided within the relevant result and legend sections.

## RESULTS

### Generation of a hα-Syn/NRF2 mouse model of Parkinson’s disease.

To comprehensively address the involvement of NRF2 in PD pathogenesis we used a cross breeding approach. To begin with, hemizygous Thy1-α-Syn (*hα-Syn^+^*) and Nrf2 knockout (Nrf2*^-/-^*) mice were bred to establish a *hα-Syn^+^/Nrf2^+/-^* strain (Fig. 1A). The *hα-Syn^+^/Nrf2^+/-^* mice were then further bred over a few generations to obtain four genotypes: (1) *hα-Syn^+^* with both endogenous copies of Nrf2 (*hα-Syn^+^/Nrf2^+/+^*), (2) *hα-Syn^+^* lacking both copies of Nrf2 (*hα-Syn^+^/Nrf2^-/-^*), (3) wild-type mice with both copies of Nrf2 (*hα-Syn^-^/Nrf2^+/+^*), and (4) wild-type mice lacking both copies of Nrf2 (*hα-Syn^-^/Nrf2^-/-^*) (Fig. 1A). Littermates from these four genotypes were used throughout the course of this study. The genotypes of all four strains were determined before the experiment with polymerase chain reaction (PCR) amplification analysis of DNA obtained from tail tissue (Fig. 1B, C), and western blot analysis (Fig. 1D). Consistent with the genotype of the mice, NRF2 was detectable only in *hα-Syn^-^/Nrf2^+/+^* and *hα-Syn^+^/Nrf2^+/+^* mice, and hα-Syn was seen only in the *hα-Syn^+^/Nrf2^-/-^* and *hα-Syn^+^/Nrf2^+/+^* mice. No immediate phenotypic changes in behavior or physical attributes were observed in the four groups of mice.

### Loss of NRF2 enhances behavioral deficits and induces TH neuron loss in the hα-Syn overexpressing mice.

**Figure 2.**
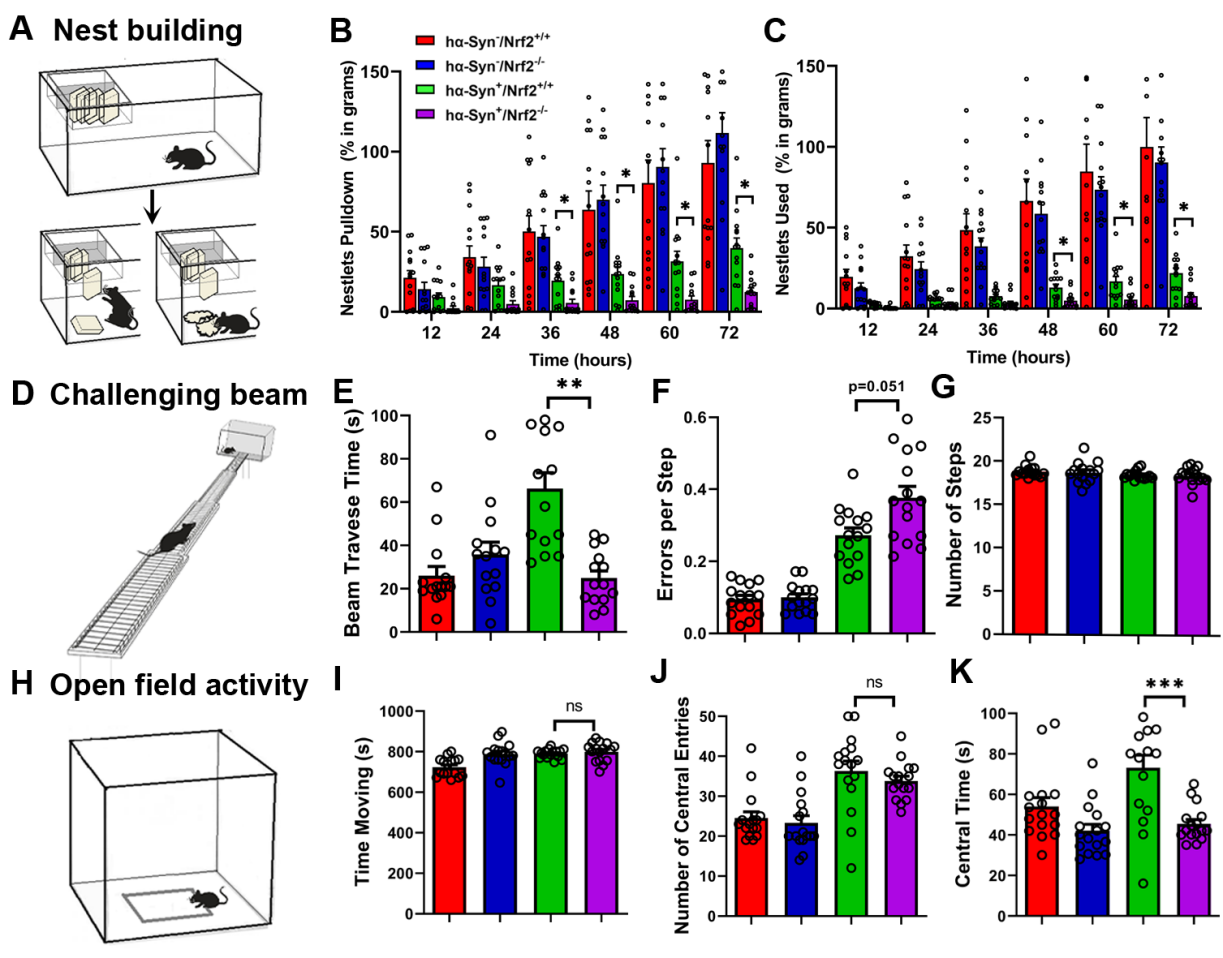
NRF2 knockout exacerbates behavioral deficits in hα-Syn^+^ mice. (A) Cartoon depiction of the nest building behavior test. (B-C) shows the amount of nestlet material pulled down and used (in grams) by 3 mos *hα-Syn^-^/Nrf2^+/+^*, *hα-Syn^-^/Nrf2^-/-^, hα-Syn^+^/Nrf2^+/+^* and *hα-Syn^+^/Nrf2^-/-^* mice at the indicated time points. (D) shows a cartoon depiction of the challenging beam task. (E-G) shows data measuring Beam traverse time (in sec, E), errors per step (F), and number of steps taken to cross the beam (G). (H) shows a cartoon of the open field activity set-up. (I-K) show data measuring the time for which the animals were moving (sec, I), number of central box entries (J), and time spent in central box (sec, K) across all four mouse strains. [*hα-Syn^-^/Nrf2^+/+^ (n=14)*, *hα-Syn^-^/Nrf2^-/-^(n=15), hα-Syn^+^/Nrf2^+/+^ (n=17)* and *hα-Syn^+^/Nrf2^-/-^ (n=15);* *p<0.05, **p<0.01, ***p<0.001, One-way ANOVA with Tukey’s post-hoc test].

To determine the effects of NRF2 loss on motor and other spontaneous behaviors, all four strains of mice were subjected to nest building, challenging beam, and open field tasks at 3 mos of age. In the nest building task, pieces of cotton nesting material (nestlets) are placed in the feeding bin at the top of the cage, and the amount of nestlet pulled down and used for nest building can be assessed (Fig. 2A). As expected, mice expressing hα-Syn (*hα-Syn^+^/Nrf2^+/+^* and *hα-Syn^+^/Nrf2^-/-^*) both pulled down (Fig. 2B) and used less (Fig. 2C) material than their non-hα-Syn expressing counterparts (Nestlet pulldown - p<0.001, F_5, 260_ = 125.54; Nestlet used - p<0.001, F_5, 260_ = 51.31, Two-way RM-ANOVA; Pairwise statistical comparisons are provided in Supplementary Table 1). However, hα-Syn overexpressing mice which lack NRF2 (*hα-Syn^+^/Nrf2^-/-^*) showed significantly reduced abilities to both pull down and use nestlets compared to hα-Syn overexpressing mice with NRF2 (*hα-Syn^+^/Nrf2^+/+^*) (Fig. 2B, C). Next, we assessed the ability of the mice to cross an increasingly narrow beam (Fig. 2D) by measuring the time taken and steps utilized to traverse the beam, as well as the number of foot slip errors. Interestingly, *hα-Syn^+^/Nrf2^+/+^* mice exhibited a slower traverse time than the *ha-Syn^-^* controls but *hα-Syn^+^/Nrf2^-/-^* showed quicker traversal across the beam (Fig. 2E; P = 0.009, F_3, 60_ = 5.305, One-way ANOVA). Moreover, *hα-Syn^+^/Nrf2^-/-^* mice also showed a higher number of errors per step than the *hα-Syn^+^/NRF2^+/+^* mice (Fig. 2F; P = 0.0519, F_3, 58_ = 33.09, One-way ANOVA). There were no significant differences in the number of steps utilized to cross the beam across all four groups (Fig. 2G). These data infer that loss of NRF2 in the hα-Syn overexpressing mice results in a more error prone hyperactive (and potentially anxious) phenotype. To further confirm a change in both locomotor function and anxiety-like behavior, an open field test, which determines spontaneous movements and entries/time spent in a central area of the field was used (Fig. 2H). While all four strains spent a similar amount of time moving in the open field arena (Fig. 2I), both *hα-Syn^+^/Nrf2^+/+^* and *hα-Syn^+^/Nrf2^-/-^* mice entered the central area more than the *hα-Syn^-^/Nrf2^+/+^* and *hα-Syn^-^/Nrf2^-/-^* mice (Fig. 2J; P = 0.0011, F_3, 60_ = 10.86, One-way ANOVA). This suggested increased novelty seeking behavior and exploration-related hyperactivity in the *hα-Syn^+^* mice, compared to *hα-Syn^-^* mice. However, *hα-Syn^+^/Nrf2^-/-^* mice spent significantly less time exploring the central field than the *hα-Syn^+^/Nrf2^+/+^* mice (Fig. 2K, Supplementary Table 1; P = 0.0002, F_3, 60_ = 8.647, One-way ANOVA) signifying greater anxiety-like behavior in the novel environment (unprotected central area of the field) in these animals. Overall, these findings indicate that a loss of NRF2 exacerbates motor dysfunction and induces affective changes in mice overexpressing hα-Syn.

**Figure 3.**
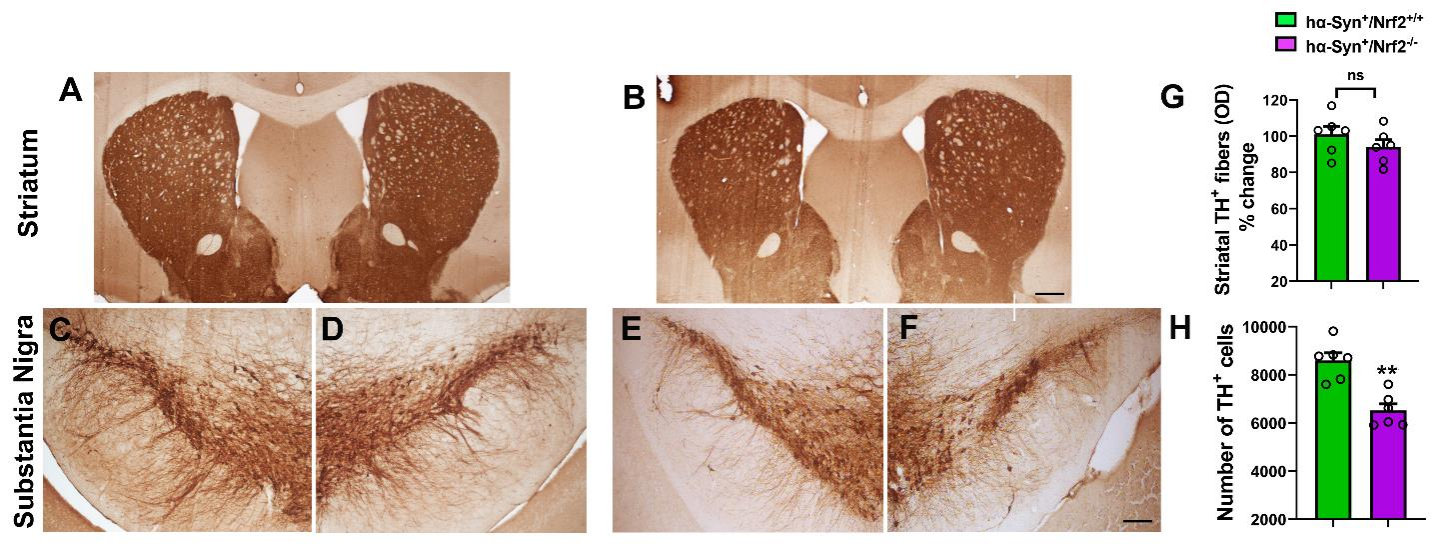
Effects of NRF2 loss on TH expression in the nigrostriatum of NRF2/α-Syn mice. Representative images of Tyrosine Hydroxylase (TH) immunostaining in the substantia nigra (SN, A-B) and striatum (ST, C-F) of *hα-Syn^+^/Nrf2^+/+^* and *hα-Syn^+^/Nrf2^-/-^* mice. (G) optical density of striatal TH^+^ fiber immunoreactivity (presented as percent of control *hα-Syn^+^/Nrf2^+/+^* values). (H) Unbiased stereological counts estimating total number of TH^+^ DA neurons in the lateral tier of the SN. *hα-Syn^+^/Nrf2^+/+^ (n=6)* and *hα-Syn^+^/Nrf2^-/-^ (n=6);* * p<0.01, unpaired t test. Scale bars = 50 μm shown in B for A-B and in F for C-F.

Given the heightened motor deficits seen in the *hα-Syn^+^/Nrf2^-/-^* mice, we examined Tyrosine Hydroxylase (TH) immunoreactivity in the substantia nigra (SN) and striatum to assess the nigrostriatal pathway. The Thy1-α-Syn mice typically do not exhibit loss of TH^+^ midbrain DA neurons in the SN [30]. However, a loss of TH immunoreactivity was evident within the lateral tier of the SN in the *hα-Syn^+^/Nrf2^-/-^* animals compared to *hα-Syn^+^/Nrf2^+/+^* controls (Fig. 3C-F). Unbiased stereological counts (via the MBF Stereoinvestigator system) indicated that *hα-Syn^+^/Nrf2^-/-^* mice indeed had significantly lower numbers of TH^+^ neurons in the SN (P = 0.007, unpaired t-test, t = 4.84, df = 10), compared to *hα-Syn^+^/Nrf2^+/+^* mice (Fig. 3H). This indicated that NRF2 loss had induced the degeneration of midbrain dopaminergic neurons. Interestingly, no notable differences in striatal TH immunoreactivity (Fig. 3A-B) between the *hα-Syn^+^/Nrf2^-/-^* and *hα-Syn^+^/Nrf2^+/+^* mice were observed (quantification in Fig. 3G).

### Oligomerization of α-Syn is promoted by genetic ablation of NRF2.

**Figure 4.**
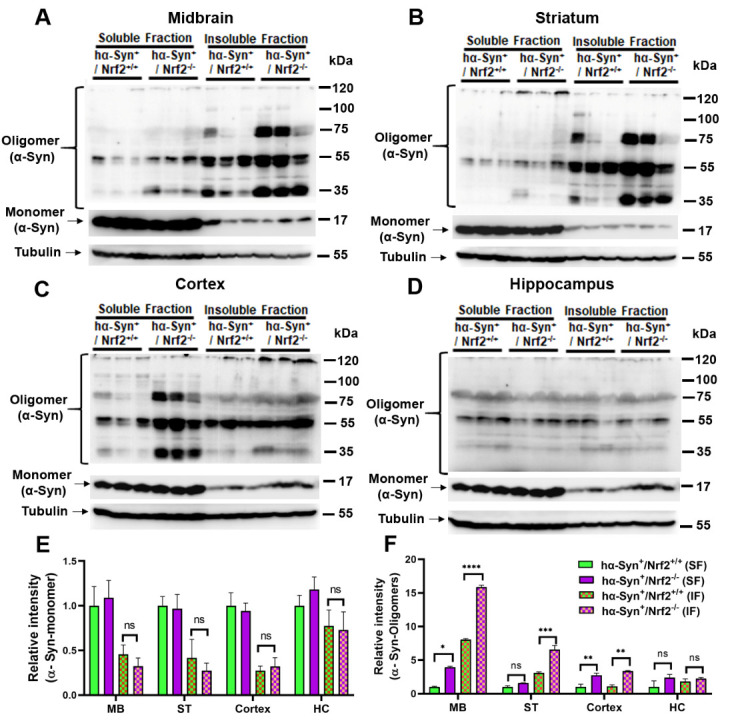
NRF2 loss induces α-Syn oligomerization. (A-D) Triton X-100 soluble and insoluble fractions were isolated from the indicated brain regions (midbrain [MB], striatum [ST], cortex and hippocampus [HC]) of 3 mos old *hα-Syn^+^/Nrf2^+/+^* and *hα-Syn^+^/Nrf2^-/-^* mice, and protein levels of monomeric and oligomeric species of α-Syn were determined by western blotting. Tubulin was used as a loading control. (E-F) shows a bar graph representing the relative densitometric quantification of α-Syn monomers and oligomers from panels (A-D). Data is represented as fold change from control. [*hα-Syn^-^/Nrf2^+/+^ (n=3)*, *hα-Syn^-^/Nrf2^-/-^(n=3), hα-Syn^+^/Nrf2^+/+^ (n=3)* and *hα-Syn^+^/Nrf2^-/-^ (n=3);* *p<0.05, **p<0.01, ***p<0.001, ****p<0.0001, One-way ANOVA with Tukey’s post-hoc test].

The oligomerization and eventual formation of insoluble α-Syn aggregates is a common pathological hallmark associated with neuronal cell dysfunction and death during PD. To determine if the behavioral deficits observed in the 3 mos old *hα-Syn^+^/Nrf2^-/-^* mice were associated with α-Syn oligomerization and aggregation, different PD-relevant brain regions were assessed for monomeric and oligomeric α-Syn species levels using western blotting of soluble and insoluble protein fractions. Specifically, four brain regions were studied; the midbrain (MB), striatum (ST), cortex, and hippocampus (HC). The expression of monomeric α-Syn was similar between hα-Syn overexpressing mice that either possessed or lacked NRF2, both within the soluble and insoluble fractions across the four brain regions (Fig. 4A-E). However, the expression of insoluble oligomeric α-Syn species were notably increased in the MB and ST of *hα-Syn^+^/Nrf2^-/-^* mice compared to *hα-Syn^+^/Nrf2^+/+^* mice (Fig. 4A, B). This observation was confirmed by densitometric analysis as shown in Fig. 4F (MB - P < 0.0001, F_3, 8_ = 158.2; ST - P = 0.0002, F_3, 8_ = 70.70; One-way ANOVA). There was also a significant increase in the expression of insoluble oligomeric α-Syn in the cortex (Fig. 4C and F; P = 0.0010, F_3, 8_ = 21.65, One-way ANOVA). Soluble oligomeric α-Syn was also seen to be higher in both the MB and cortex (Fig. 4A, C, F; MB - P < 0.0001, F_3, 8_ = 158.2; Cortex - P = 0.001, F_3, 8_ = 21.65; One-way ANOVA). There was no difference in the expression levels of soluble or insoluble α-Syn in the HC between the two genotypes (Fig. 4D-F). Thus, loss of NRF2 causes greater α-Syn oligomerization and formation of insoluble aggregates in the MB, ST, and cortex, but not in the HC, of the 3 mos old hα-Syn overexpressing mice.

### NRF2 loss increases phospho-α-Syn levels.

**Figure 5.**
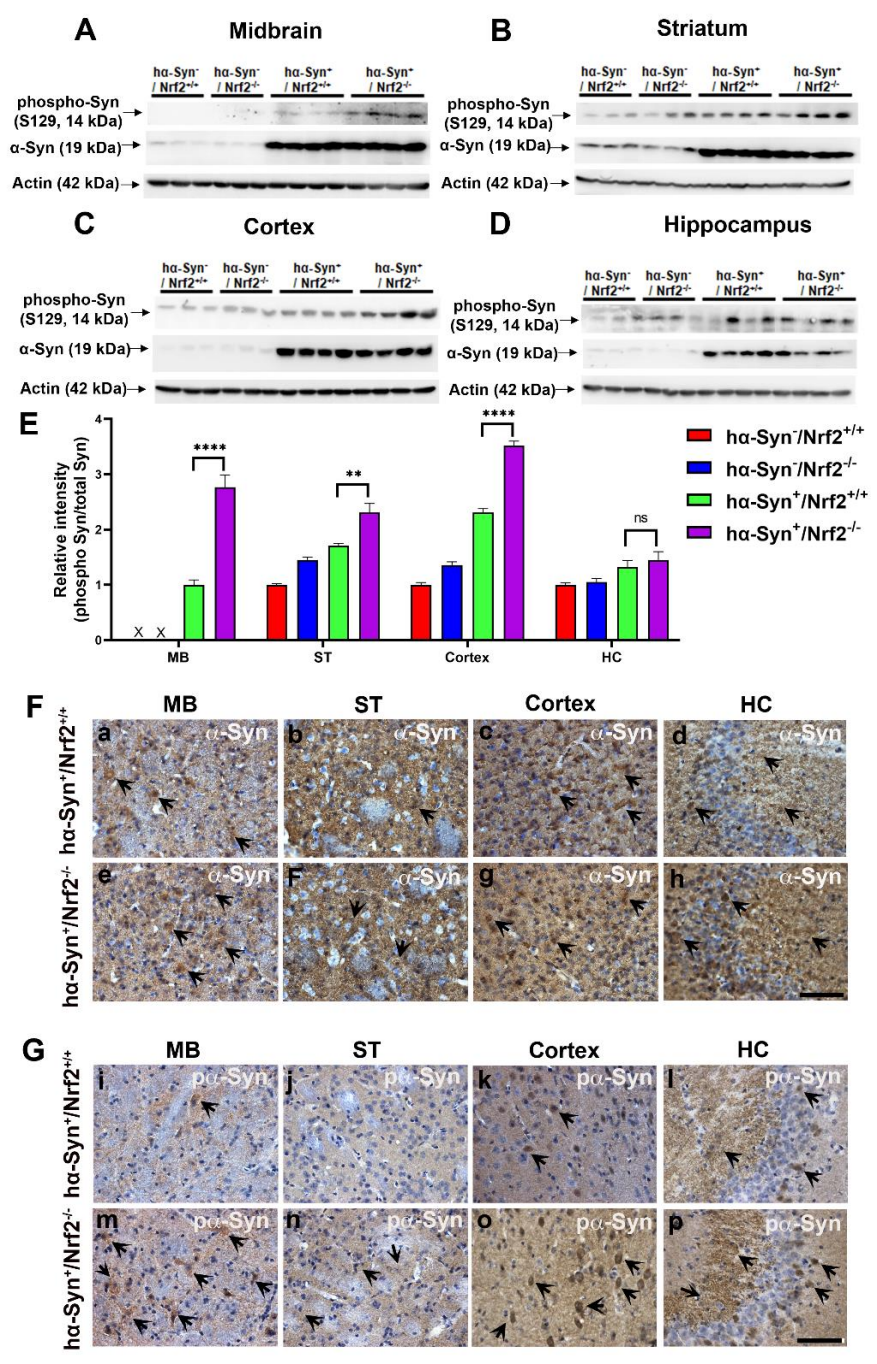
Phosphorylation of α-Syn is increased in NRF2 knockout mice overexpressing hα-Syn. (A-D) α-Syn and phospho-Syn levels were evaluated by WB in the midbrain (MB), striatum (ST), cortex and hippocampus (HC) of 3 mos old *hα-Syn^-^/Nrf2^+/+^*, *hα-Syn^-^/Nrf2^-/^*, *hα-Syn^+^/Nrf2^+/+^*, and *hα-Syn^+^/Nrf2^-/-^* mice. (E) shows a bar graph quantifying phosphorylated α-Syn oligomers normalized to α-Syn monomer using densitometry. Actin was used as a loading control. Data is represented as fold change from the indicated control. (F) has representative images of α-Syn immunohistochemical staining in the midbrain (MB), striatum (ST), cortex and hippocampus (HC) of *hα-Syn^+^/Nrf2^+/+^* and *hα-Syn^+^/Nrf2^-/-^* mice (a-h). (G) includes representative images of phospho-Syn immunohistochemical staining in the midbrain (MB), striatum (ST), cortex and hippocampus (HC) of *hα-Syn^+^/Nrf2^+/+^* and *hα-Syn^+^/Nrf2^-/-^* mice (i-p). [*hα-Syn^-^/Nrf2^+/+^ (n=3)*, *hα-Syn^-^/Nrf2^-/-^(n=3), hα-Syn^+^/Nrf2^+/+^ (n=4)* and *hα-Syn^+^/Nrf2^-/-^ (n=4);* **p<0.01, ****p<0.0001, One-way ANOVA with Tukey’s post-hoc test]. Scale bar = 50 μm, is shown in h for a-h; in p for i-p.

Post-translational modification of α-Syn, particularly phosphorylation and oxidative alterations, plays a significant role in the oligomerization and eventual aggregation of α-Syn into insoluble Lewy bodies. To determine the mechanism by which loss of NRF2 was increasing α-Syn aggregation, α-Syn phosphorylation status was assessed in the MB, ST, cortex, and HC of the 3 mos old mice of all the four genotypes. As expected, western blot analysis of S129 phosphorylation showed generally greater levels of phospho-α-Syn, as well as total synuclein, in all four brain regions in hα-Syn overexpressing mice than mice lacking hα-Syn (Fig. 5A-D). The data also indicated that phospho-α-Syn levels were higher in the MB, ST and cortex of *hα-Syn^+^/Nrf2^-/-^* mice than *hα-Syn^+^/Nrf2^+/+^* animals (Fig 5A-C). Densitometric analysis revealed that the ratio of phospho/total α-Syn was higher in the MB, ST, and cortex of *hα-Syn^+^/Nrf2^-/-^* mice compared to *hα-Syn^+^/Nrf2^+/+^* controls (Fig. 5E; MB - P < 0.0001, F_3, 10_ = 91.16; ST - P = 0.0053, F_3, 10_ = 29.35; One-way ANOVA). No differences were found in the hippocampus.

Immunohistochemistry was also conducted to analyze α-Syn (Fig. 5F [a-h]) and phospho-α-Syn (Fig. 5G [i-p]) expression *in situ* at the morphological level. Data showed comparable α-Syn immunoreactivity across all four regions of *hα-Syn^+^/Nrf2^+/+^* (Fig. 5F [a-d]) and *hα-Syn^+^/Nrf2^-/-^* mice brains (Fig. 5F [e-h]). However, higher phospho-α-Syn immunoreactivity was observed in the MB, ST, and cortex of *hα-syn^+^/Nrf2^-/-^* mice (Fig. 5G [i-p]). More phospho-α-Syn labeled cells, as well as greater extracellular phospho-α-Syn, was noted in the *hα-Syn^+^/Nrf2^-/-^* mice (arrows in Fig. 5G [m-p]) compared to *hα-Syn^+^/Nrf2^+/+^* controls (Fig. 5G [i-l]). No particular differences in phospho-α-Syn expression were noted in the hippocampus between the *hα-Syn^+^/Nrf2^-/-^* mice and *hα-Syn^+^/Nrf2^+/+^* mice (Fig. 5G [l, p]). High magnification imaging of cells in the different regions showed a mostly cytosolic distribution of phospho-α-Syn, with some nuclear localization (Supplementary Fig. 1A-H). These data, along with the data in Figure 4, indicate that the loss of NRF2 promotes α-Syn phosphorylation and subsequent oligomerization.

**Figure 6.**
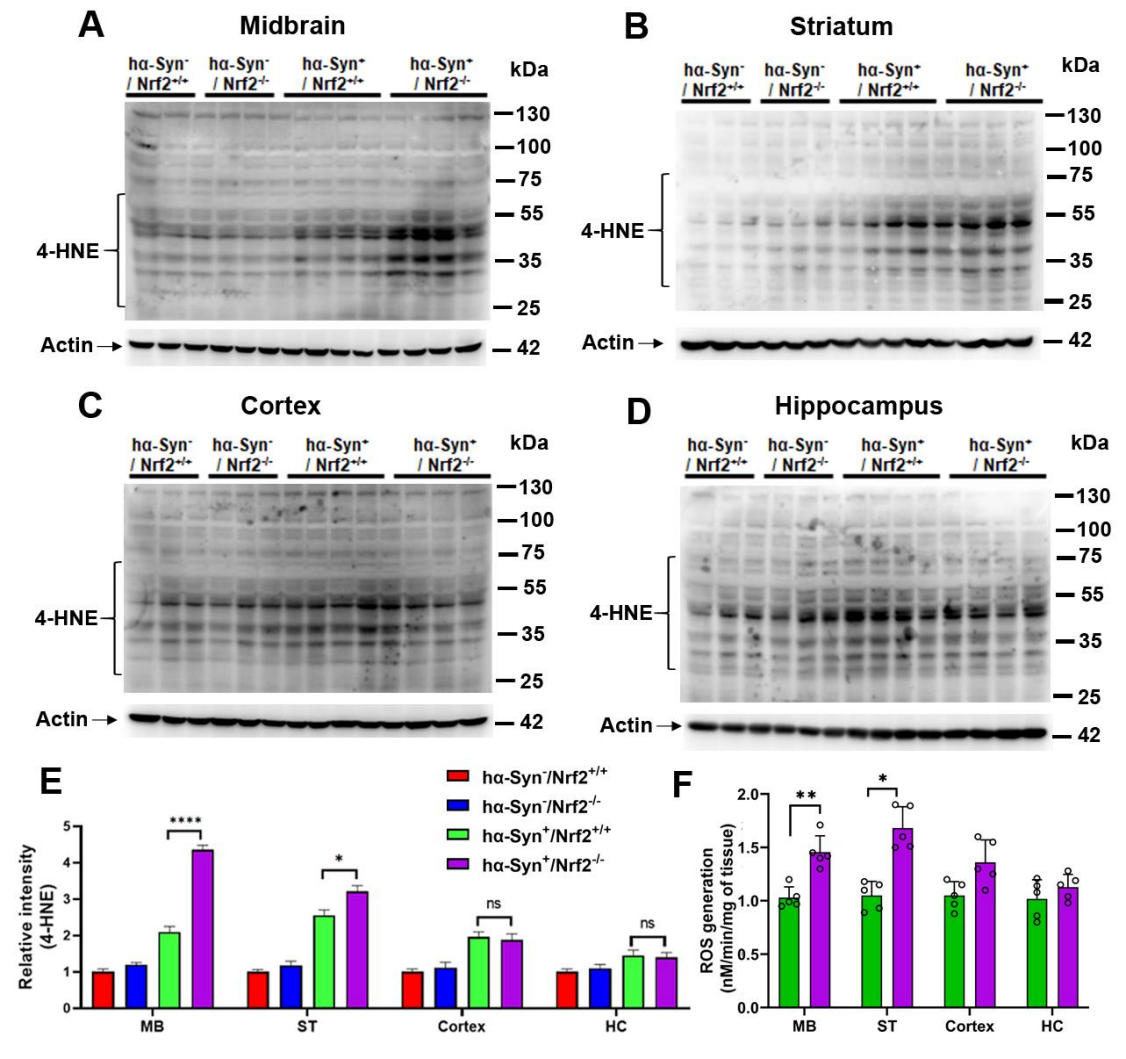
NRF2 knockout mice overexpressing α-Syn exhibit increased basal oxidative stress. (A-D) depicts levels of 4-Hydroxynonenal (4-HNE)-adduct formation, an indicator of increased oxidative stress, in the midbrain (MB), striatum (ST), cortex and hippocampus (HC) of 3 mos old *hα-Syn^-^/Nrf2^+/+^*, *hα-Syn^-^/Nrf2^-/-^*, *hα-Syn^+^/Nrf2^+/+^*, and *hα-Syn^+^/Nrf2^-/-^* mice via western blotting. Actin is used as a loading control. (E) shows a bar graph representing the densitometric quantification of 4-HNE adducts in the indicated brain regions. Data is represented as fold change from the indicated control. [*hα-Syn^-^/Nrf2^+/+^ (n=3)*, *hα-Syn^-^/Nrf2^-/-^(n=3), hα-Syn^+^/Nrf2^+/+^ (n=4)* and *hα-Syn^+^/Nrf2^-/-^ (n=4*); **p<0.01, ****p<0.0001, One-way ANOVA with Tukey’s post-hoc test]. (F) has data on ROS generation which was measured by electron paramagnetic resonance (EPR) spectroscopy in the midbrain (MB), striatum (ST), cortex and hippocampus (HC) of *hα-Syn^+^/Nrf2^+/+^* and *hα-Syn^+^/Nrf2^-/-^* mice. Data are represented as nM/min/mg of tissue and normalized to control. [*hα-Syn^-^/Nrf2^+/+^ (n=5)*, *hα-Syn^-^/Nrf2^-/-^(n=5), hα-Syn^+^/Nrf2^+/+^ (n=5)* and *hα-Syn^+^/Nrf2^-/-^ (n=5);* *p<0.05, unpaired *t*-test].

To specifically examine phospho-α-Syn expression in relation to midbrain TH-positive neurons within the SN, we also conducted double immunostaining for TH and phospho-α-Syn (Supplementary Fig. 1I-Q). In terms of phospho-α-Syn, higher expression was seen in the hα-Syn^+^/Nrf2^-/-^ mice (Supplementary Fig. 1M-O) than hα-Syn^+^/Nrf2^+/+^ controls (Supplementary Fig. 1I-K). Interestingly, neurons with high phospho-α-Syn expression appeared to have lower or lost their TH immunoreactivity (arrows in Supplementary Fig. 1O, high mag views in [P]). These results were further confirmed by quantification of TH^+^/phospho-α-Syn^+^ cells in the SN (Supplementary Fig. 1Q; P = 0.0049, unpaired t-test, t = 5.638, df = 4). These data suggested that increased phospho-α-Syn was potentially contributing to nigral neuronal toxicity/dysfunction.

### α-Syn overexpressing mice develop higher oxidative stress levels in the absence of NRF2.

As NRF2 is known to regulate numerous aspects of the antioxidant response, the effect of genetic ablation of NRF2 on oxidative stress was studied. 4-hydroxynonenal (4-HNE), a reactive lipid species that accumulates in the brain during PD progression, can form adducts with protein cysteine residues and serve as an indicator of increased oxidative modification of proteins. Overall, both the hα-Syn overexpressing mouse strains, *hα-Syn^+^/Nrf2^+/+^* and *hα-Syn^+^/Nrf2^-/-^* mice, showed an increase in 4-HNE protein adduct formation in the MB, ST, cortex, and HC compared to the non hα-Syn overexpressing mouse strains, *hα-Syn^-^/Nrf2^+/+^* and *hα-Syn^-^/Nrf2^-/-^* (Fig. 6A-D). Interestingly when *hα-Syn^+^/Nrf2^-/-^* mice were compared to *hα-Syn^+^/Nrf2^+/+^* mice, it was observed that NRF2 loss had promoted significantly higher 4-HNE adduct levels in the MB and ST (Fig. 6A-B). 4-HNE adduct levels in the cortex and HC were similar in the *hα-Syn^+^/Nrf2^-/-^* mice and *hα-Syn^+^/Nrf2^+/+^* mice (Fig. 6C-D). Quantification via densitometry confirmed these data (Fig. 6E; MB - P < 0.0001, F_3, 10_ = 172.2; ST - P = 0.021, F_3, 10_ = 60.92; Cortex - P = 0.97, F_3, 10_ = 11.33; HC - P = 0.9, F_3, 10_ = 2.837, One-way ANOVA). Oxidative stress in the hα-Syn overexpressing mice was further analyzed using electron paramagnetic resonance spectroscopy (Fig. 6F). Here, higher amounts of reactive oxygen species (ROS) were found in the MB and ST of *hα-Syn^+^/Nrf2^-/-^* mice than in *hα-syn^+^/Nrf2^+/+^* controls (Fig. 6F; MB - P = 0.0402, unpaired t-test, t = 2.446, df = 8; ST - P= 0.0378, unpaired t-test, t = 2.486, df = 8). These results indicate that NRF2 loss had significantly enhanced oxidative stress in hα-Syn overexpressing mice especially in the MB and ST at 3 mos of age.

### Cellular and molecular markers of neuroinflammation rise with NRF2 loss

**Figure 7.**
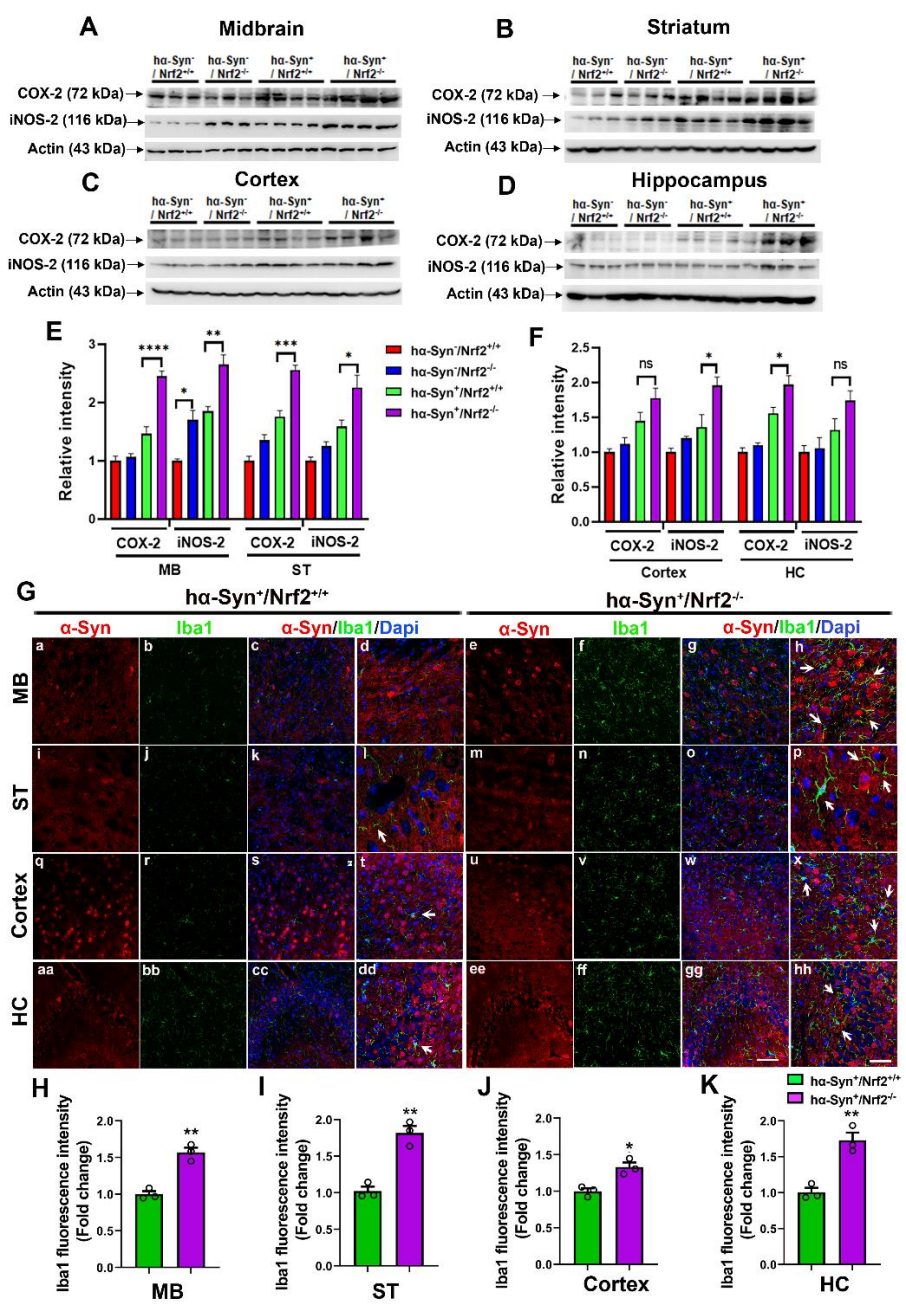
Inflammatory markers are elevated in NRF2 knockout mice overexpressing hα-Syn. (A-D). Expression of the inflammatory markers COX-2 and iNOS-2 as determined by western blotting in the midbrain (MB), striatum (ST), cortex and hippocampus (HC) of 3 mos old *hα-Syn^-^/Nrf2^+/+^*, *hα-Syn^-^/Nrf2^-/-^*, *hα-Syn^+^/Nrf2^+/+^*, and *hα-Syn^+^/Nrf2^-/-^* mice. Actin was used as a loading control. (E-F) has a bar graph representing the densitometric quantification of COX-2 and iNOS-2 protein levels from the indicated brain regions. Data is represented as fold change from the indicated control. [*hα-Syn^-^/Nrf2^+/+^ (n=3)*, *hα-Syn^-^/Nrf2^-/-^(n=3), hα-Syn^+^/Nrf2^+/+^ (n=4)* and *hα-Syn^+^/Nrf2^-/-^ (n=4)*; *p<0.05, **p<0.01, ***p<0.0001 ****p<0.0001, One-way ANOVA with Tukey’s post-hoc test]. (G) shows representative images of α-Syn and Iba1 IHC staining in midbrain (MB), striatum (ST), cortex and hippocampus (HC) from *hα-Syn^+^/Nrf2^+/+^* and *hα-Syn^+^/Nrf2^-/-^* mice (a-hh). Scale bar = 25 μm for a-c, e-g, i-k, m-o, q-s, u-w, aa-cc, ee-gg is shown in gg; Scale bar = 10 μM for d, h, l, p, t, x, dd and hh is in hh. (H-K) show the quantification of the Iba1 signal in the midbrain (MB), striatum (ST), cortex and hippocampus (HC). Data are presented as fold change from control (*hα-Syn^+^/Nrf2^+/+^* values). [*hα-Syn^+^/Nrf2^+/+^ (n=3)* and *hα-Syn^+^/Nrf2^-/-^ (n=3);* *p<0.05, Unpaired t-tests]

α-Syn accumulation has been linked with the chronic inflammation observed in many PD patients. Along with its well-established antioxidant role, NRF2 has also been shown to act as an important anti-inflammatory mediator. As such, mice lacking NRF2 would be expected to exhibit a more pro-inflammatory phenotype in the presence of excess α-Syn. To test this, protein levels of cyclooxygenase-2 (COX-2) and inducible nitric oxide synthase-2 (iNOS-2), which are induced during inflammation, were studied via western blot analysis. As expected, COX-2 and iNOS-2 levels were both higher in the MB, ST, cortex, and HC of the hα-Syn overexpressing mouse strains (*hα-Syn^+^/Nrf2^+/+^* and *hα-Syn^+^/Nrf2^-/-^*) compared to the *hα-Syn^-^* controls (Fig. 7A-D). Moreover, *hα-Syn^+^/Nrf2^-/-^* mice had higher overall levels of both inflammatory proteins in relation to their counterpart *hα-Syn^+^/Nrf2^+/+^* mice in the MB, ST and cortex with no significant differences seen in the HC (Fig. 7E-F; MB [COX-2] - P < 0.0001, F_3, 10_ = 49.36, [iNOS2] P = 0.0041, F_3, 10_ = 27.14; ST - [COX-2] - P = 0.0003, F_3, 10_ = 55.48, [iNOS2] P = 0.025, F_3, 10_ = 14.44; Cortex - [COX-2] - P = 0.2029, F_3, 10_ = 9.157, [iNOS2] P = 0.0245, F_3, 10_ = 10.55; HC - [COX-2] - P = 0.039, F_3, 10_ = 21.68, [iNOS2] P =0.189, F_3, 10_ = 5.545, One-way ANOVA). To further assess neuroinflammation in the hα-Syn overexpressing mice with or without NRF2, we examined microglial activation in the four brain regions in these animals (Fig. 7G). Interestingly, increased numbers of ionized calcium binding adaptor 1 (Iba1) positive microglial cells (green), were noted in all four regions in the NRF2 knockout animals (Fig. 7G f, n, v, ff) compared to controls (Fig. 7G b, j, r, bb). Also, this increased microglial presence was seen in areas with greater α-Syn immunostaining (Fig. 7G [h, p, x, hh]; Syn in red, arrows point to microglia in green) in *hα-Syn^+^/Nrf2^-/-^* mice. Quantification of Iba1 fluorescence intensity in the MB (Fig. 7H), ST (Fig. 7I), cortex (Fig.7J) and hippocampus (Fig.7K) confirmed these findings. (Fig. 7H-K; MB- P = 0.0018, unpaired t-test, t = 7.3664, df = 4; ST- P = 0.002, unpaired t-test, t = 6.991, df = 4; cortex- P = 0.01, unpaired t-test, t = 4.438, df = 4, HC- P = 0.004, unpaired t-test, t = 5.886, df = 4). Overall, these data support the notion that NRF2 suppresses the inflammatory cascade initiated by hα-Syn overexpression.

**Figure 8.**
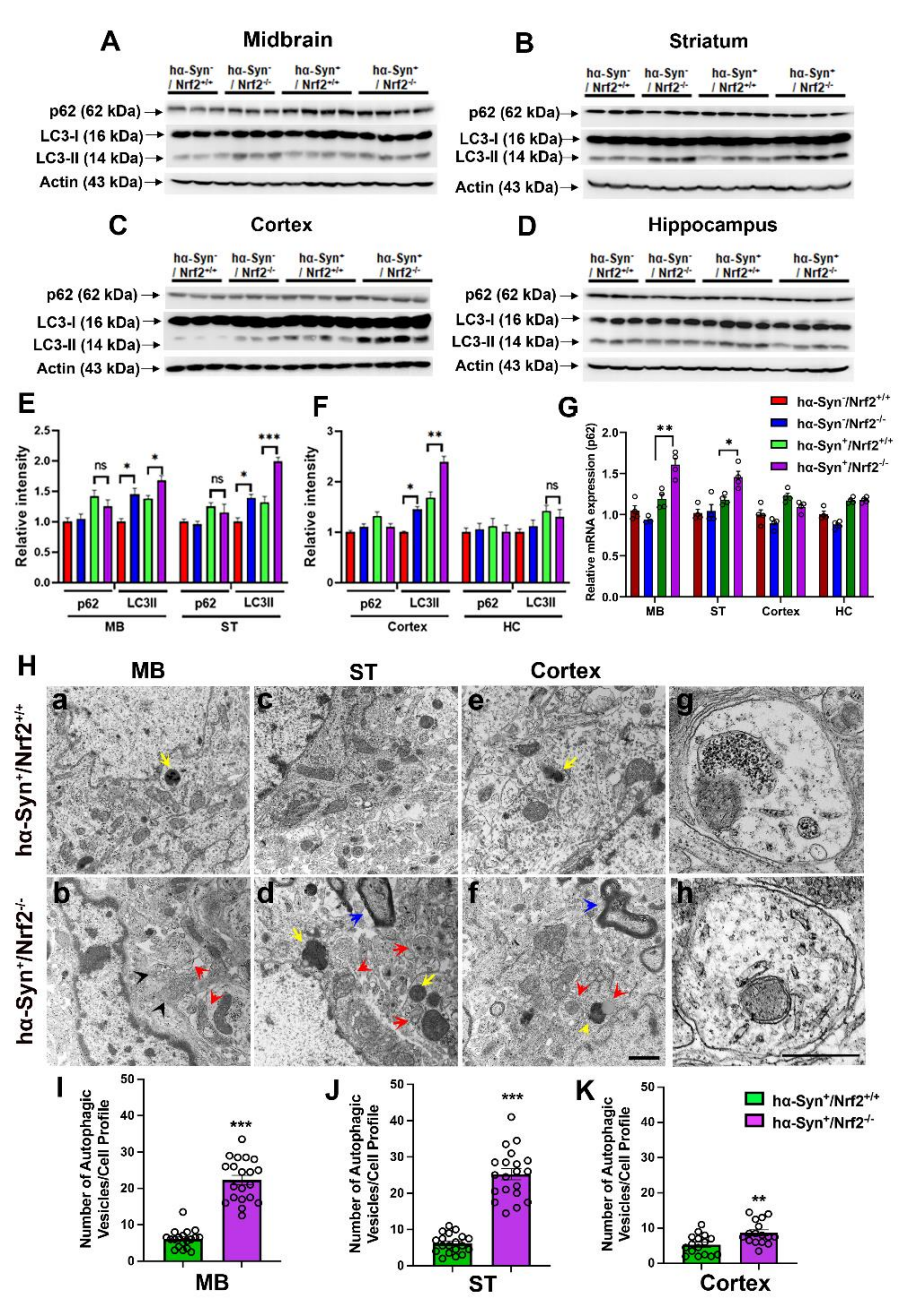
Autophagic function is altered in hα-Syn mice with NRF2 deficiency. (A-D) shows protein levels of the key autophagy markers p62 and LC3 II as determined by western blotting in the midbrain (MB), striatum (ST), cortex and hippocampus (HC) of 3 mos old *hα-Syn^-^/Nrf2^+/+^*, *hα-Syn^-^/Nrf2^-/-^*, *hα-Syn^+^/Nrf2^+/+^*, and *hα-Syn^+^/Nrf2*^-/-^ mice. Actin was used as a loading control. (E-F) depicts bar graphs representing the relative densitometry quantification of p62 and LC3 II in the indicated brain regions. Data is represented as fold change from the indicated control. [*hα-Syn^-^/Nrf2^+/+^ (n=3)*, *hα-Syn^-^/Nrf2^-/-^(n=3), hα-Syn^+^/Nrf2^+/+^ (n=4)* and *hα-Syn^+^/Nrf2^-/-^ (n=4)*]. (G) shows mRNA levels of p62 in the indicated brain regions. [*hα-Syn^-^/Nrf2^+/+^ (n=4)*, *hα-Syn^-^/Nrf2^-/-^(n=4), hα-Syn^+^/Nrf2^+/+^ (n=4)* and *hα-Syn^+^/Nrf2^-/-^ (n=4)*]. [*p<0.05, **p<0.01, ***p<0.0001, One-way ANOVA with Tukey’s post-hoc test]. (H) shows representative transmission electron micrographs of midbrain (MB), striatum (ST), and cortex of 3 mos old *hα-Syn^+^/Nrf2^+/+^* and *hα-Syn^+^/Nrf2^-/-^* mice (a-f), and high magnification views of a typical autophagosome (g) and autolysosome (h). Red arrows point to autophagosome- and autolysosome-like structures; yellow arrows indicate dense residual bodies; blue arrows point to multilamellar bodies (MLBs); black arrows show multivesicular bodies (MVBs). Quantification of autophagic vesicles is in I-K. Data are presented as average number of autophagic vacuoles/cell profile across 16-20 cell profiles (*hα-Syn^+^/Nrf2^+/+^ (n=2)* and *hα-Syn^+^/Nrf2^-/-^ (n=2)*; **p<0.01, ***p<0.0001, Unpaired t-tests]. Scale bar = 500 nm for a-f is shown in f, and g-h is shown in h.

### Loss of NRF2 induces autophagic changes in hα-Syn overexpressing mice.

Autophagic dysfunction is a key feature of PD that is known to drive the accumulation of toxic α-Syn aggregates. NRF2 has also been shown to regulate key components of the autophagy machinery [6, 22]. Thus, we evaluated autophagy by assessing protein levels of two standard autophagy markers, LC3-II and SQSTM1/p62 through western blot. LC3-II is generated by the conjugation of cytosolic LC3-I to phosphatidyl-ethanolamine (PE) on the surface of nascent autophagosomes (thus it is an autophagosome marker), and p62 is involved in binding and recruiting ubiquitinated cargo for autophagy-mediated degradation (p62 itself is an autophagy substrate). LC3-II levels were significantly higher in the MB, ST, and cortex of the *hα-Syn^+^/Nrf2^-/-^* mice than *hα-Syn^+^/Nrf2^+/+^* animals (Fig. 8A-C & E-F; MB - P = 0.0443, F_3, 10_ = 14.62; ST - P = 0.0004, F_3, 10_ = 28.63; Cortex - P = 0.0013, F_3, 10_ = 35.00, One-way ANOVA). Interestingly, higher LC3-II levels were also noted in the MB, ST and cortex of *hα-Syn^-^/Nrf2^-/-^* mice when compared to *hα-Syn^-^/Nrf2^+/+^*(Fig. 8A-C & E-F). No specific differences in LC3-II were noted in the hippocampus between the four mice groups (Fig. 8D-F). In terms of p62 expression, there were no significant differences between the four groups in any of the brain regions (Fig. 8A-B & E-F). Nevertheless, qRT-PCR indicated that p62 transcript levels were higher in *hα-Syn^+^/Nrf2^-/-^* mice than *hα-Syn^+^/Nrf2^+/+^* controls (Fig. 8G; MB - P = 0.056, F_3, 12_ = 20.36; ST - P = 0.006, F_3, 12_ = 22.91, One-way ANOVA). These data, along with the LC3-II changes, indicated increased basal autophagy in the *hα-Syn^+^/Nrf2^-/-^* mice. To further understand these autophagic changes, we conducted transmission electron microscopy on tissues from the MB, ST and cortex. Here, it was observed that *hα-Syn^+^/Nrf2^-/-^* mice had more autophagic vesicles (arrows) in these 3 regions than *hα-Syn^+^/Nrf2^+/+^* mice (Fig. 8H; quantification in Fig. 8I-K) (MB - P <0.0001, unpaired t-test, t = 11.18, df = 37; ST - P <0.0001, unpaired t-test, t = 11.44, df = 37; Cortex - P = 0.003, unpaired t-test, t = 3.2, df = 30). Typical double membraned autophagosomes and autolysosomes (red arrows in Fig. 8H [b, d, f]; high magnification images of an autophagosome and autolysosome are in Fig. 8H [g & h]), as well as what appeared to be dense residual bodies (yellow arrows), multilamellar bodies (MLBs, blue arrows), and multivesicular bodies (MVBs, black arrows), which are all vesicular compartments of the autophagic pathway, were noted in the *hα-Syn^+^/NRF2^-/-^* mice. These data indicate that depletion of NRF2 results in a compensatory increase in basal autophagy, plausibly to cope with the greater load of insoluble α-Syn aggregates.

## DISCUSSION

In essence, our study generates novel data that reveal that an early lack of NRF2 promotes α-Syn proteinopathy and other hallmark PD pathologies such as DA neuron degeneration, oxidative stress, inflammation and altered autophagy. Importantly, the study also determines that NRF2 loss exacerbates motor deficits and induces anxiety, underlining the functional relevance of NRF2 activity in PD. These results emphasize a vital role for NRF2 in the pathogenesis of PD.

Our data show that the lack of NRF2 worsens motor function and induces anxiety in the α-Syn overexpressing mice. Specifically, deficits in the nest building, challenging beam, and open field tasks were seen. The nest building task involves a combination of rearing and complex fine motor skills to grasp and pull-down cotton from the feeder bin. Moreover, on the floor of the cage, animals require the use of orofacial and forelimb movements to tear apart the nesting material and incorporate it into their bedding. It was found that both the amount of cotton retrieved from the bin, as well as the amount of cotton shredded for nest building, was lower for the NRF2 knockout mice at all tested time points, with significant differences from control noted between 36 and 72 hrs. This indicated that NRF2 loss promoted impairments in both fine and gross motor function in the animals. In the challenging beam task, interestingly, the *hα-Syn^+^/Nrf2^-/-^* mice traversed the beam faster but committed more foot slip errors than the *hα-Syn^+^/Nrf2^+/+^* animals, suggesting reduced motor coordination and a hyperactive phenotype. Assessment in the open field showed that although both the NRF2 knockout and wild-type animals overexpressing α-Syn entered the central box equally, the NRF2 knockout mice spent less time in the box. Mice typically exhibit ‘thigmotaxis’ (hugging the walls) relative to exploring the central, more unprotected area of the open field [41]. Thus, the increased central entries suggest higher novelty seeking behavior and exploration-related hyperactivity in the *hα-Syn^+^* mice, compared to *hα-Syn^-^* animals. However, the reduced time spent in the center (more thigmotaxis) in the *hα-Syn+* mice lacking NRF2, compared to those with NRF2, may indicate strong emotional responses to the novel environment in these animals (that is greater anxiety-like behavior in the exposed central area of the field). All in all, these data indicate that a deficit in NRF2 promotes motor and affective changes reminiscent of symptoms in individuals at early stages of PD [42-45], and provide the first evidence for a direct role for NRF2 in the expression of PD-relevant behaviors.

We studied the distribution and expression of α-Syn in four brain regions, specifically the striatum, midbrain, cortex and hippocampus. It was found that the lack of the NRF2 gene does not significantly alter monomeric α-Syn expression, however, levels of phosphorylated α-Syn were increased in the striatum, midbrain and cortex. An elevation in oligomeric and insoluble α-Syn species was also determined in these regions. These data infer that NRF2 loss enhances α-Syn phosphorylation, which causes oligomerization and eventual conversion of α-Syn into insoluble aggregates in the hα-Syn overexpressing mice. These findings align with a study by Lastres-Becker et al., which showed that virally induced expression of α-Syn in the ventral midbrain of NRF2 knockout mice resulted in the increased immunohistochemical expression of phosphorylated α-Syn and greater α-Syn aggregation in the neurites of dopaminergic neurons [25]. Nevertheless, in contrast, our studies provide a detailed qualitative and quantitative analysis of different α-Syn isoforms across multiple brain regions in a translationally relevant model where there is continual endogenous production of human α-Syn. Moreover, our studies link the molecular alterations in α-Syn to behavioral (motor and emotional) changes in the *hα-Syn^+^/Nrf2^-/-^* mice. Additionally, connected to the synuclein alterations, we also noted higher basal autophagy in the *hα-Syn^+^/Nrf2^-/-^* mice. These data suggest that the buildup of α-Syn aggregates due to the enhanced modification of monomers and formation of oligomers in the brains of *hα-Syn^+^/NRF2^-/-^* mice activated autophagy and may overwhelm clearance through the autophagosome-lysosome machinery leading to neuronal toxicity [46]. Supporting this notion, a notable reduction in DA neuron numbers was observed in the SN of *hα-Syn^+^/NRF2^-/-^* mice. Moreover, TH-positive neurons in the SN with high phospho-α-Syn expression appeared to have lower TH immunoreactivity in the *hα-Syn^+^/Nrf2^-/-^* mice, suggesting that α-Syn aggregation could be contributing to TH neuron dysfunction.

Elevated oxidative stress and neuroinflammation were observed in the NRF2 deficient hα-Syn overexpressing animals. In particular, higher oxidative stress levels (4-HNE adducts and ROS) were found in the midbrain and striatum of *hα-Syn^+^/NRF2^-/-^* mice compared to controls, with associated pro-inflammatory changes involving increased COX2 and iNOS levels in the midbrain, striatum and cortex. Additionally, more microglia were also observed especially in α-Syn rich regions in mice lacking NRF2. In this context, it has long been recognized that activated microglia surround Lewy bodies in postmortem PD brains [47-50]. α-Syn, which is a major constituent of Lewy bodies, has been shown to bind to and activate microglia through toll-like receptors (TLRs) that mediate innate immunity [30, 51, 52]. Such activated microglia not only represent an immunological reaction to ongoing damage by phagocytosing damaged cells and debris, but can also emit harmful ROS and pro-inflammatory cytokines [47]. In turn, these oxidative stress and pro-inflammatory changes can further aggravate α-Syn misfolding and aggregation [46, 53, 54]. Interestingly, the Lastres-Becker et al., study also reported increased gliosis and inflammation in NRF2 knockout mice in response to acute viral expression of α-Syn as well as impaired phagocytotic ability of microglia in NRF2 deficient animals [25]. Given NRF2’s classical role in modulating the antioxidant and anti-inflammatory response, the stimulation of oxidative stress and inflammation in the *hα-Syn^+^/Nrf2^-/-^* is not completely unexpected [7, 55]. However, the significantly higher intensity of these changes in *hα-Syn^+^/Nrf2^-/-^* as compared to their wild-type counterparts and *hα-Syn^-^/Nrf2^-/-^* animals suggests an important role for NRF2 in controlling α-Syn related chronic inflammation and excessive production of damaging free radical species, further solidifying the importance of maintaining proper NRF2 levels even during the earliest stages of neurodegeneration. This finding is also of particular interest as it indicates that premature loss of NRF2 could result in a more rapid and severe progression of the disease.

Interestingly, the hippocampus was the least affected region in our studies. It has been shown in the Thy1-hα-Syn mice that hippocampal changes in terms of α-synuclein expression and cognitive alterations are consistently seen only after 4-5 mos of age [30]. Addressing such regional differences due to NRF2 loss with age will be important to investigate in future studies. With regards to cell-specific effects, it has been reported that astrocytes express high levels of NRF2 and may be the major cell type for NRF2 activation during neurodegeneration. For example, in the basal ganglia of 1-methyl-4-phenyl-1,2,3,6 tetrahydropyridine hydro-chloride (MPTP)-treated mice, astrocytes are the main cell type to upregulate NRF2 in response to sulforaphane (an NRF2 inducer) treatment [56]. Moreover, NQO1, peroxiredoxin 6, and HO-1, all NRF2 targets, are strongly expressed in astrocytes in SN of human post-mortem PD brains [8, 56, 57]. Additionally, it has been shown that the viral overexpression of NRF2 in astrocytes renders mice resistant to MPTP toxicity and mutant α-Synuclein [24, 58], and that the transplantation of NRF2 overexpressing astrocytes protects against 6-hydroxydopamine (6-OHDA) neurotoxicity [59]. Nevertheless, on the other hand, it is also known that nuclear NRF2 expression is largely restricted to neurons in the human post-mortem brain SN of PD patients [10], and HO-1 is also increased [8]. Furthermore, increased NRF2 expression seen in response to dimethyl fumarate treatment (NRF2 inducer) in MPTP-treated mice is partially localized to midbrain neurons [60]. Overall, these findings highlight a key involvement of astrocytes, and also yet unknown interactions between neurons and astrocytes that may be crucial to NRF2’s role in PD pathogenesis. Such cell-specific effects related to NRF2 will be vital to understand.

In summary, this study reveals the early loss of NRF2 as a critical driver of α-Syn-mediated pathology and cellular dysfunction that leads to behavioral deficits similar to those commonly observed in PD patients. Indeed, NRF2 may be crucial to PD pathogenesis through its effects on core neurodegenerative processes such as oxidative stress, protein quality control and inflammation that ultimately induce cell death. By extension, a deeper understanding of NRF2-based mechanisms will support the development of much needed therapeutics for mitigating the harmful neuropathological features across all stages of this devastating degenerative disease.

## Supplementary Materials

The Supplementary data can be found online at: www.aginganddisease.org/EN/10.14336/AD.2021.0511.


