# Erratum to “NRF2 Loss Accentuates Parkinsonian Pathology and Behavioral Dysfunction in Human α-Synuclein Overexpressing Mice”

**DOI:** 10.14336/AD.2023.10917

**Published:** 2024-05-07

**Authors:** Annadurai Anandhan, Nhat Nguyen, Arjun Syal, Luke A Dreher, Matthew Dodson, Donna D. Zhang, Lalitha Madhavan

In our article “NRF2 Loss Accentuates Parkinsonian Pathology and Behavioral Dysfunction in Human α-Synuclein Overexpressing Mice” published in the July 2021 issue of Aging and Disease [Aging Dis. 2021 Jul 1;12(4):964-982], we have noted some inadvertent errors. In Figure 4C, the western blot image of the a-synuclein monomer band, was mistakenly duplicated to Figure 4D. In Figure 7, the same b-actin band relevant to figure 8B, was also incorrectly presented in Figure 7C. We have attached the correct Figures 4 and 7. The errors do not change the conclusions of the article. The authors truly apologize for the errors and the inconvenience caused.

**Figure 4.**
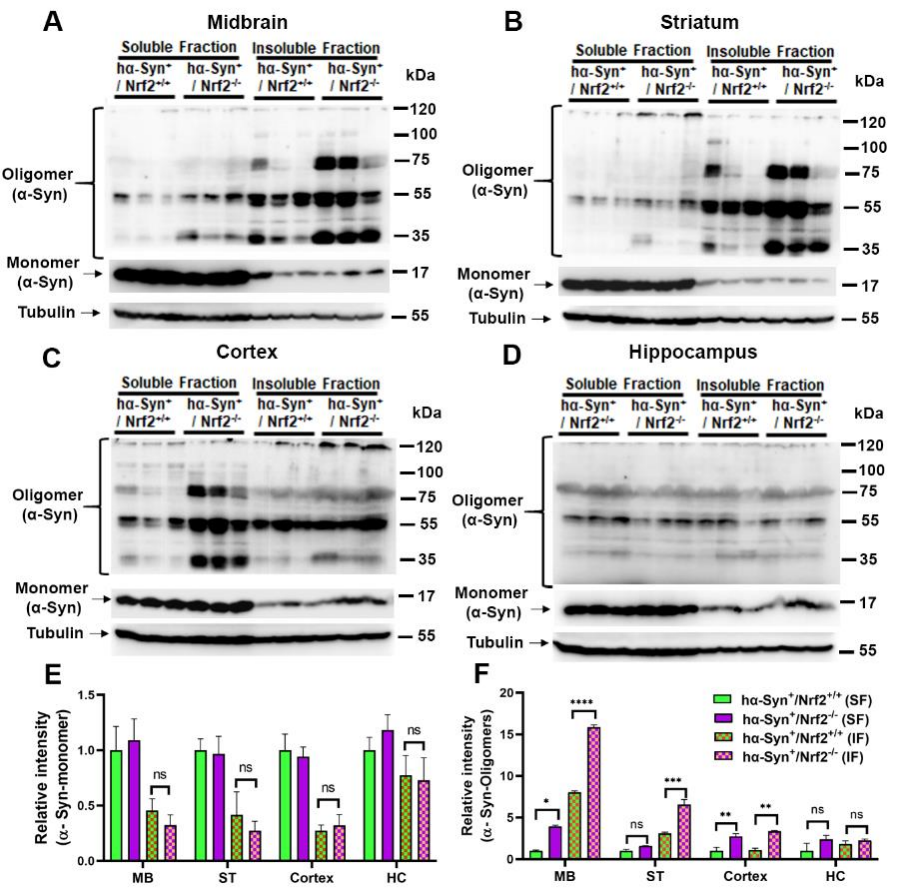
**NRF2 loss induces α-Syn oligomerization**. (**A-D**) Triton X-100 soluble and insoluble fractions were isolated from the indicated brain regions (midbrain [MB], striatum [ST], cortex and hippocampus [HC]) of 3 mos old *hα-Syn+/Nrf2+/+* and *hα-Syn+/Nrf2-/-* mice, and protein levels of monomeric and oligomeric species of α-Syn were determined by western blotting. Tubulin was used as a loading control. (**E-F**) shows a bar graph representing the relative densitometric quantification of α-Syn monomers and oligomers from panels (A-D). Data is represented as fold change from control. [*hα-Syn-/Nrf2+/+ (n=3)*, *hα-Syn-/Nrf2-/-(n=3), hα-Syn+/Nrf2+/+ (n=3)* and *hα-Syn+/Nrf2-/- (n=3);* *p<0.05, **p<0.01, ***p<0.001, ****p<0.0001, One-way ANOVA with Tukey’s post-hoc test].

**Figure 7.**
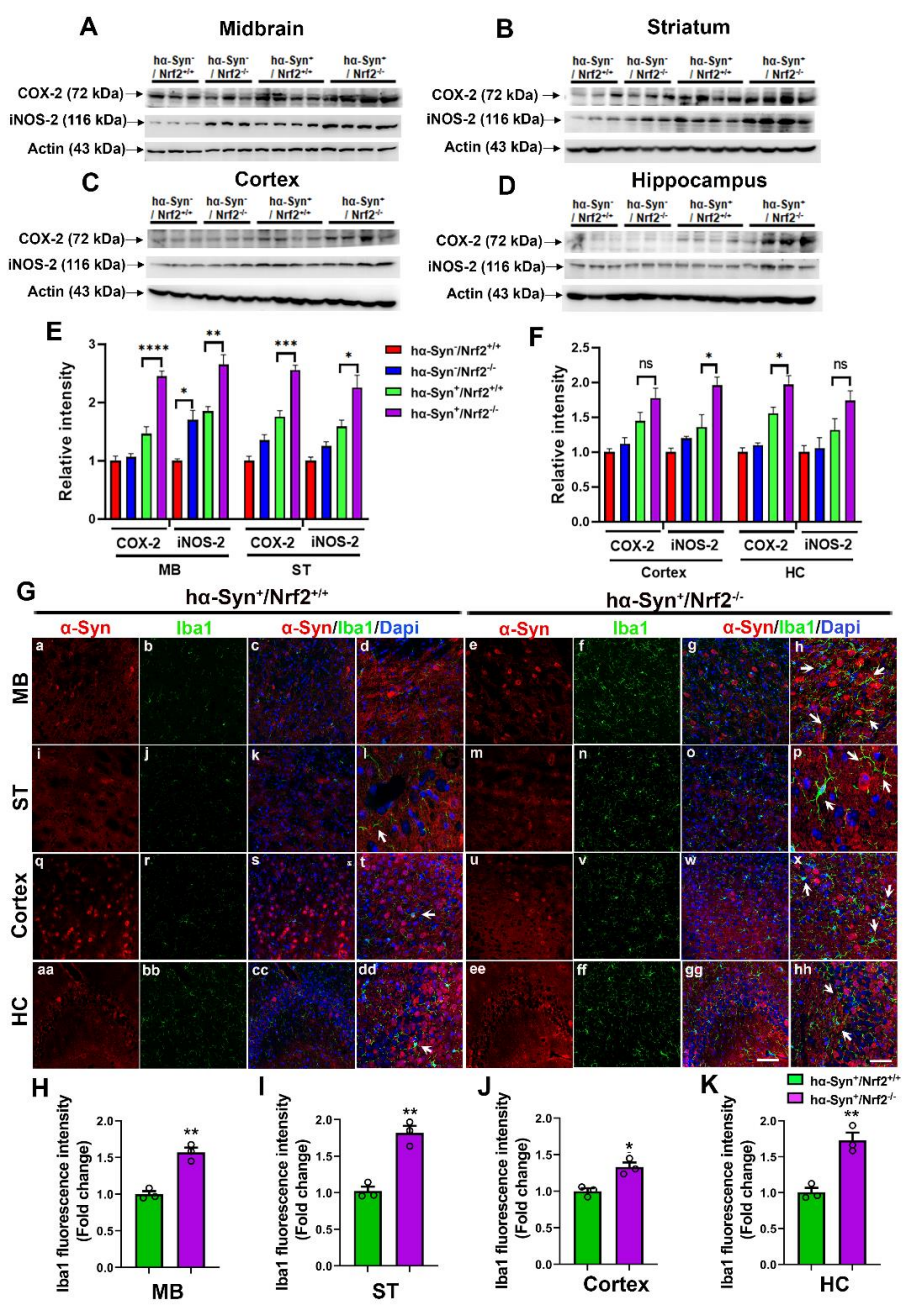
**Inflammatory markers are elevated in NRF2 knockout mice overexpressing hα-Syn**. (**A-D**). Expression of the inflammatory markers COX-2 and iNOS-2 as determined by western blotting in the midbrain (MB), striatum (ST), cortex and hippocampus (HC) of 3 mos old *hα-Syn-/Nrf2+/+*, *hα-Syn-/Nrf2-/-*, *hα-Syn+/Nrf2+/+*, and *hα-Syn+/Nrf2-/-* mice. Actin was used as a loading control. (**E-F**) has a bar graph representing the densitometric quantification of COX-2 and iNOS-2 protein levels from the indicated brain regions. Data is represented as fold change from the indicated control. [*hα-Syn-/Nrf2+/+ (n=3)*, *hα-Syn-/Nrf2-/-(n=3), hα-Syn+/Nrf2+/+ (n=4)* and *hα-Syn+/Nrf2-/- (n=4)*; *p<0.05, **p<0.01, ***p<0.0001 ****p<0.0001, One-way ANOVA with Tukey’s post-hoc test]. (**G**) shows representative images of α-Syn and Iba1 IHC staining in midbrain (MB), striatum (ST), cortex and hippocampus (HC) from *hα-Syn+/Nrf2+/+* and *hα-Syn+/Nrf2-/-* mice (a-hh). Scale bar = 25 μm for a-c, e-g, i-k, m-o, q-s, u-w, aa-cc, ee-gg is shown in gg; Scale bar = 10 μM for d, h, l, p, t, x, dd and hh is in hh. (**H-K**) show the quantification of the Iba1 signal in the midbrain (MB), striatum (ST), cortex and hippocampus (HC). Data are presented as fold change from control (*hα-Syn+/Nrf2+/+* values). [*hα-Syn+/Nrf2+/+ (n=3)* and *hα-Syn+/Nrf2-/- (n=3);* *p<0.05, Unpaired t-tests]

