# Supplementary material for: NRF2 Loss Accentuates Parkinsonian Pathology and Behavioral Dysfunction in Human α-Synuclein Overexpressing Mice
Source: Aging Dis. 2021 Jul 1;12(4):964–82. doi: 10.14336/AD.2021.0511 (PMC8219498; doi:10.14336/AD.2021.0511)
Supplement: Supplementary file 1 [file AD-12-4-964-s.pdf]

SUPPLEMENTARY DATA

NRF2 Loss Accentuates Parkinsonian Pathology and Behavioral Dysfunction in Human α-Synuclein Overexpressing Mice

Annadurai Anandhan[1, 2], Nhat Nguyen[3], Arjun Syal[4], Luke A Dreher[5], Matthew Dodson[1], Donna D Zhang[1*], Lalitha Madhavan[2,6*]

© 2021. Anandhan A et al. Published online at http://www.aginganddisease.org/EN/10.14336/AD.2021.0510

SUPPLEMENTARY DATA

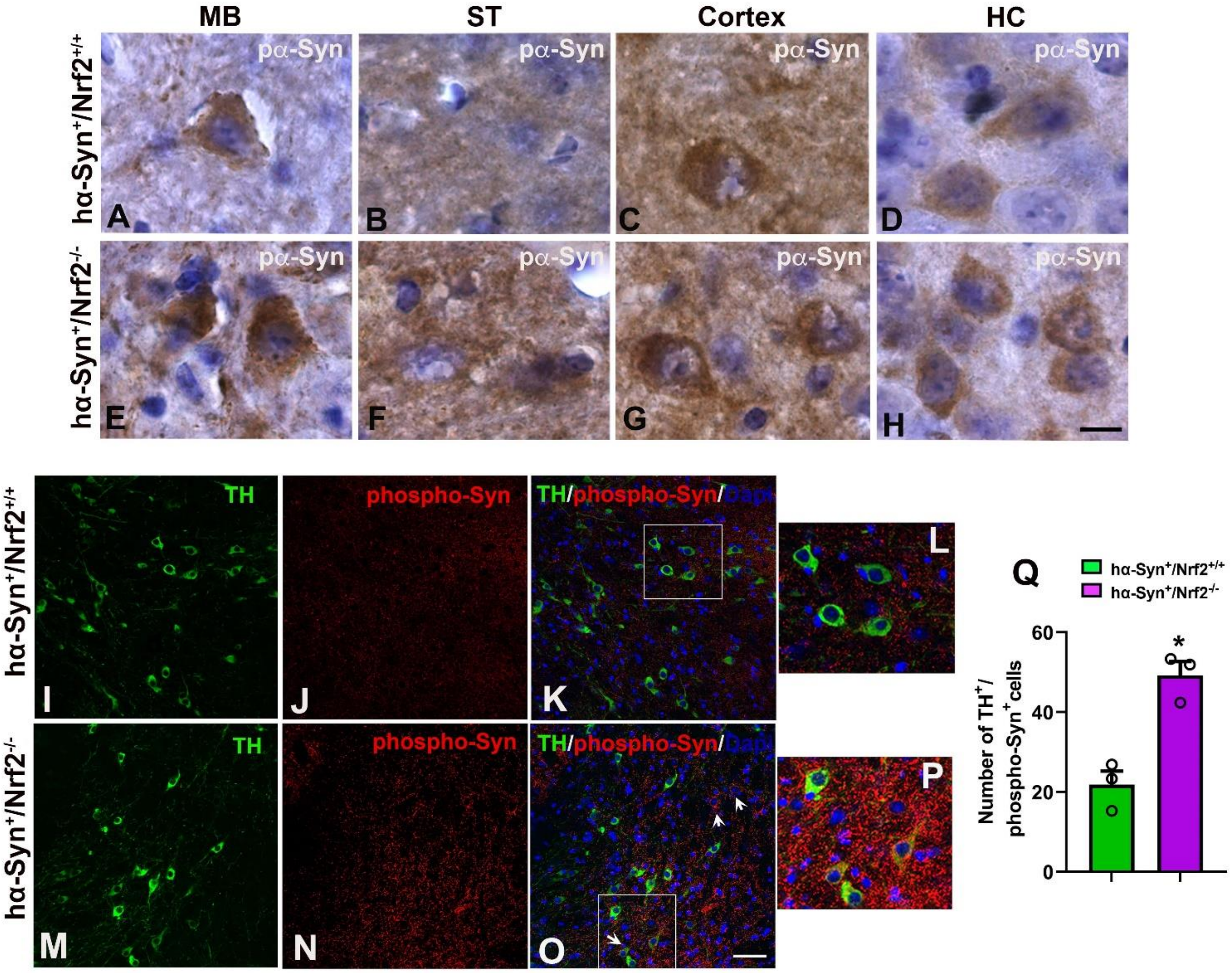

Supplementary Figure 1. Characterization of phospho-α-Syn immunoreactivity in relation to SN TH neurons of NRF2/α-Syn mice. High magnification images showing subcellular localization of phospho-α-Syn in the MB (**A, E**), ST (**B, F**), Cortex (**C, G**), and HC (**D, H**) of $h\alpha\text{-}Syn^{+}/Nrf2^{+/+}$ and $h\alpha\text{-}Syn^{+}/Nrf2^{-/-}$ mice. Representative images of phospho-α-Syn immunofluorescence staining in relation to TH neurons in the SN of $h\alpha\text{-}Syn^{+}/Nrf2^{+/+}$ (**I-L**) and $h\alpha\text{-}Syn^{+}/Nrf2^{-/-}$ mice (**M-P**). **L** and **P** are high magnification views of areas in **K and O**. Arrows in O point to cells with high phospho-α-Syn but low TH immunoreactivity. (**I**) shows the quantification of TH^{+}/phospho-Syn^{+} cells in the SN (*n=3*). [*p<0.05, Unpaired t-test]. Scale bar = 10 μm for A-H is in H; Scale bar = 25 μm for I-K & M-O is in G.

© 2021. Anandhan A et al. Published online at http://www.aginganddisease.org/EN/10.14336/AD.2021.0510

SUPPLEMENTARY DATA

Supplementary Table 1. Statistical details of pairwise comparisons via two-way RM ANOVA in the Nest building behavioral task.

hα-Syn^{+}/Nrf2$^{+/+}$* vs *hα-Syn^{+}/Nrf2$^{-/-}$						
	Nestlet pulldown			***Nestlet used***		
	t value	p value	Significant?	t value	p value	Significant?
**12 hrs**	0.73	1.0	No	0.016	1.0	No
**24 hrs**	1.06	1.0	No	0.218	1.0	No
**36 hrs**	2.72	0.048	Yes	0.37	1.0	No
**48 hrs**	2.84	0.034	Yes	3.7	0.002	Yes
**60 hrs**	2.94	0.026	Yes	2.69	0.04	Yes
**72 hrs**	2.82	0.036	Yes	2.72	0.044	Yes

© 2021. Anandhan A et al. Published online at http://www.aginganddisease.org/EN/10.14336/AD.2021.0510
